# Synthesis of 5′-Thiamine-Capped RNA

**DOI:** 10.3390/molecules25235492

**Published:** 2020-11-24

**Authors:** Marvin Möhler, Katharina Höfer, Andres Jäschke

**Affiliations:** Institute of Pharmacy and Molecular Biotechnology, Heidelberg University, Im Neuenheimer Feld 364, 69120 Heidelberg, Germany; marvin.moehler@uni-heidelberg.de (M.M.); Katharina.Hoefer@synmikro.mpi-marburg.mpg.de (K.H.)

**Keywords:** RNA modification, non-canonical initiating nucleotide (NCIN), in vitro transcription, thiamine (vitamin B1), thiamine adenosine triphosphate, thiamine-adenosine diphosphate, thiamine-capped RNA, chemical capture of thiamine-capped RNA, click chemistry

## Abstract

RNA 5′-modifications are known to extend the functional spectrum of ribonucleotides. In recent years, numerous non-canonical 5′-modifications, including adenosine-containing cofactors from the group of B vitamins, have been confirmed in all kingdoms of life. The structural component of thiamine adenosine triphosphate (thiamine-ATP), a vitamin B1 derivative found to accumulate in *Escherichia coli* and other organisms in response to metabolic stress conditions, suggests an analogous function as a 5′-modification of RNA. Here, we report the synthesis of thiamine adenosine dinucleotides and the preparation of pure 5′-thiamine-capped RNAs based on phosphorimidazolide chemistry. Furthermore, we present the incorporation of thiamine-ATP and thiamine adenosine diphosphate (thiamine-ADP) as 5′-caps of RNA by T7 RNA polymerase. Transcripts containing the thiamine modification were modified specifically with biotin via a combination of thiazole ring opening, nucleophilic substitution and copper-catalyzed azide-alkyne cycloaddition. The highlighted methods provide easy access to 5′-thiamine RNA, which may be applied in the development of thiamine-specific RNA capture protocols as well as the discovery and confirmation of 5′-thiamine-capped RNAs in various organisms.

## 1. Introduction

Ribonucleic acid (RNA) obtains remarkable structural and functional versatility through the combination of the four canonical ribonucleosides adenosine (A), guanosine (G), cytidine (C) and uridine (U). Numerous additional modifications occur internally as well as terminally, at the 3′‑ and 5′-end, and fine-tune the functional spectrum of RNA. Such modifications can, e.g., increase the stability of RNA against degradation processes, extend the catalytic activity of ribozymes, promote RNA interactions with other molecules or assume various regulatory roles within the cellular environment [1,2,3,4,5,6].

Transcribed RNA is generally provided with a triphosphate group at the 5′-terminus. In eukaryotes, a post-transcriptional modification of messenger RNA (mRNA) with a 7‑methylguanosine (m7G) cap takes place [7,8]. The m7G cap and similar structures provide increased stability of mRNA against 5′‑exonucleolytic degradation [9,10] and facilitate the formation of the translation initiation complex crucial for protein synthesis [11,12]. For a long time, the prevailing opinion on 5′‑modification of RNAs was its exclusive existence on the eukaryotic level. Nowadays, also numerous prokaryotic 5′‑modifications have been reported and found their way into biological textbooks [4,5,13].

In 2009, the cofactors nicotinamide adenine dinucleotide (NAD) and 3′‑dephospho-coenzyme A (dephospho‑CoA) were reported to decorate the 5′‑end of RNA in *Escherichia coli* and *Streptomyces venezuelae* [14]. In vitro transcription experiments confirmed the acceptance of such adenosine-containing cofactors by RNA polymerases as non-canonical initiating nucleotides (NCINs) [15,16]. The modification levels of RNA with NAD, dephospho‑CoA and other NCINs such as flavin adenine dinucleotide (FAD) and uridine diphosphate *N*-acetylglucosamine (UDP-GlcNAc) vary considerably between organisms, which supports the hypothesis of a regulated cap epitranscriptome [17]. While liquid chromatography-mass spectrometry (LC-MS)-based methods allow for both identification and quantification of the 5′-modifications, they are not capable of determining the identity of the 5′-modified RNA sequences.

For this purpose, our group developed a chemoenzymatic capture protocol, NAD captureSeq, which allowed for the enrichment of 5′-NAD-capped RNA from *E. coli* total RNA and its analysis via next-generation sequencing (NGS) [18,19]. Particularly abundant NAD-bearing sequences were found amongst regulatory RNAs, and it was confirmed that the NAD‑cap, similar to eukaryotic modifications, increases the stability of the modified RNA [18]. An adaptation of the aforementioned protocol also led to the detection of NAD-capped RNA sequences in Gram-positive bacteria, such as *Bacillus subtilis* and *Staphylococcus aureus* [20,21], as well as in eukaryotes, like *Saccharomyces cerevisiae*, *Arabidopsis thaliana* and *Homo sapiens* [22,23,24,25]. In addition, the existence of enzymes that specifically target 5′-NAD-, dephospho-CoA- or FAD-capped RNAs, such as the nudix hydrolase NudC and proteins of the DXO family, supports the idea of regulated capping and decapping processes [24,26,27].

The highlighted B‑group vitamin coenzymes, which are incorporated into RNA as 5′-caps, stand out, in particular. With their structural properties and catalytic potential, they are discussed as an important part of the RNA world hypothesis [28,29]. NAD, FAD and dephospho‑CoA already carry an adenosine within their structure, which qualifies them as NCINs. Other members of the B vitamins conserved different structural information, suggesting their proximity to nucleic acids, such as the pyrimidine ring of thiamine (vitamin B1) [28].

Interestingly, a natural thiamine adenine dinucleotide was discovered in 2007 [30], which will in the following be referred to as thiamine adenosine triphosphate (thiamine-ATP, ThATP). The commonly known thiamine derivatives are thiamine hydroxide (ThOH) and its phosphorylated forms thiamine monophosphate (ThMP), the co-enzymatically active thiamine pyrophosphate (ThDP) and thiamine triphosphate (ThTP) [31,32,33]. Similar to thiamine triphosphate, thiamine-ATP was described as a signaling molecule due to its accumulation in *E. coli* in response to certain metabolic stress conditions, primarily during glucose starvation [30,34]. The enzymatic synthesis and hydrolysis of thiamine adenine dinucleotides were characterized [35] and thiamine-ATP was detected in other organisms as well [30,36]. A role similar to that of alarmones, which are incorporated into RNA as protective 5′-caps under cellular stress conditions [37,38], might thus also be assigned to thiamine-ATP as a potential RNA 5′-modification. This hypothesis suggests a completely unknown physiological role of thiamine.

Herein, we report the preparation and analysis of 5′-thiamine-capped RNA. A novel synthesis route for thiamine-ATP via imidazolide-based activation of phosphate groups was developed and found to be superior to the literature methodology [39]. Furthermore, the acceptance of thiamine-ATP as NCIN by T7 RNA polymerase was demonstrated. To our knowledge, this is the first communication of the enzymatic incorporation of the naturally occurring thiamine-ATP as a 5′‑cap of RNA, which strongly suggests the existence of 5′‑thiamine-capped RNAs in vivo. Moreover, chemical modification of the 5′‑cap with biotin allowed for separation of in vitro transcribed 5′‑thiamine RNA from 5′-triphosphate RNA. Conclusively, our demonstrated methods for the preparation of 5′‑thiamine RNA will allow for the development of a thiamine-specific capture protocol and, potentially, for the discovery of 5′‑thiamine-capped RNAs in *E. coli* and other organisms.

## 2. Results

For the characterization of the newly discovered thiamine-ATP in 2007, Bettendorff and coworkers used the condensation reaction of ThDP and 5’-adenosine monophosphate (5’‑AMP) with *N*,*N*’-dicyclohexylcarbodiimide (DCC) for the preparation of the adenosine-containing thiamine derivative [30,39]. However, only small amounts of thiamine-ATP were yielded and losses occurred, particularly during several purification steps. Jessen and coworkers improved this initial synthesis using phosphordiamidites in a four-step reaction protocol, including the treatment with trifluoroacetic acid and *meta*-chloroperoxybenzoic acid [40]. Still, they were confronted with the formation of side products by homodimerization of the substrates.

To improve yields and facilitate purification, we initially decided on a two-step approach based on the reaction of ThDP with an activated 5′‑AMP. In this context, 5′‑phosphoroimidazolides of canonical nucleosides have been extensively studied. Prominent applications include the use of adenosine 5′‑phosphoroimidazolide (ImpA) and other imidazolide-activated molecules for adenylation and capping of single nucleotides or RNA sequences [41,42,43,44,45,46], for example in the original NAD captureSeq protocol [18,19].

**Figure 1:**
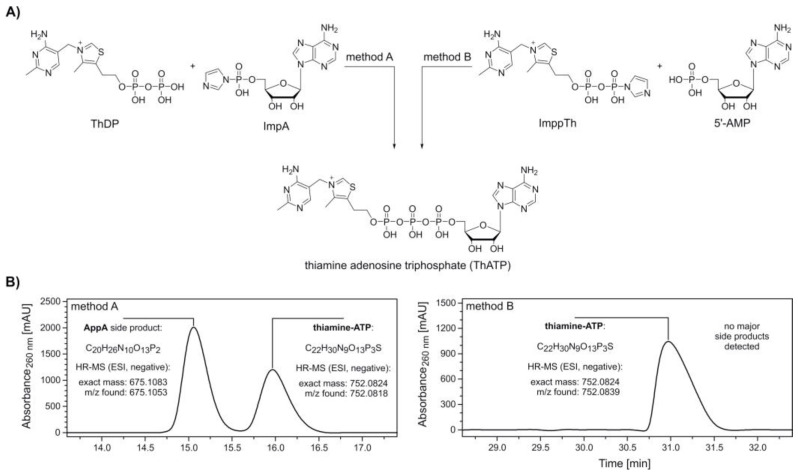
Synthesis and purification of thiamine-ATP (ThATP). (**A**) Synthesis scheme of ThATP via coupling of adenosine 5’-phosphoroimidazolide (ImpA) (method A) and thiamine diphosphate β-*P*‑imidazolide (ImppTh) (method B) to thiamine pyrophosphate (ThDP) and adenosine 5’-monophosphate (5′‑AMP), respectively. Method A: 1. ThDP, magnesium chloride (MgCl_2_), dimethylformamide (DMF), room temperature (rt), 2. ImpA; method B: 1. 5′-AMP, MgCl_2_, DMF, rt, 2. ImppTh. (**B**) High-performance liquid chromatography (HPLC, see Supplementary Figure S1) and high-resolution mass spectrometry (HR-MS) analysis confirm the formation of ThATP following both methods. The formation of the major side product *P*^1^,*P*^2^-di(adenosine-5′)-diphosphate (AppA) by homodimerization is avoided in method B.

The synthesis of thiamine-ATP was carried out following two methods (Figure 1A, method A and B), by either using an activated adenosine (ImpA) or thiamine component (thiamine diphosphate β-*P*‑imidazolide, ImppTh).

ImpA was synthesized adapting standard protocols [41,44] with slight modifications in stoichiometry and reaction times. The precipitated sodium salt of ImpA was washed several times with acetone and diethyl ether and recovered by centrifugation in 96.4% yield.

ImppTh was prepared in a similar fashion. The activation of ThDP was notably slower in comparison to 5′‑AMP. However, the imidazolide-activated ImppTh was obtained in 83.7% yield with high purity. High-performance liquid chromatography (HPLC) analysis of this new compound showed a substantial deactivation through hydrolysis in aqueous solution. Within 2 and 24 h of storage in buffered solution (0.1 M triethylammonium-acetate, pH 7.0) at room temperature, 10.0% and 37.3% of ImppTh were degraded to ThDP (Supplementary Figure S2). This process is suggested to occur even faster in non‑buffered solution, as observed during NMR analysis.

After pre‑incubation of ThDP with anhydrous MgCl_2_, ImpA was added to yield thiamine-ATP (Figure 1A, method A). HPLC analysis and electrospray ionization-mass spectrometry (ESI-MS) measurements of collected peaks confirmed the formation of thiamine-ATP in an approximate 0.7:1 ratio with *P*^1^,*P*^2^-di(adenosine-5′)-diphosphate (AppA) as a single, major side product (Figure 1B, method A and Supplementary Figure S1A).

In a similar fashion, thiamine adenosine diphosphate (thiamine-ADP, ThADP) was prepared by the reaction of ImpA with ThMP in the presence of MgCl_2_ (Supplementary Figure S3A). For this synthesis comprising the more reactive monophosphate of thiamine, thiamine-ADP was yielded in a ratio of 24:1 with AppA and eluted earlier as the side product with the given HPLC conditions (Supplementary Figures S1B and S3B).

Due to challenging separation of thiamine-ATP and AppA, a synthesis route via ImppTh was developed. Here, 5′‑AMP was pre‑incubated with MgCl_2_ before addition of ImppTh (Figure 1A, method B). The reaction yielded thiamine-ATP free from any major side products and enabled the semi-preparative purification by HPLC in a larger scale (Figure 1B, method B and Supplementary Figure S1C).

**Figure 2:**
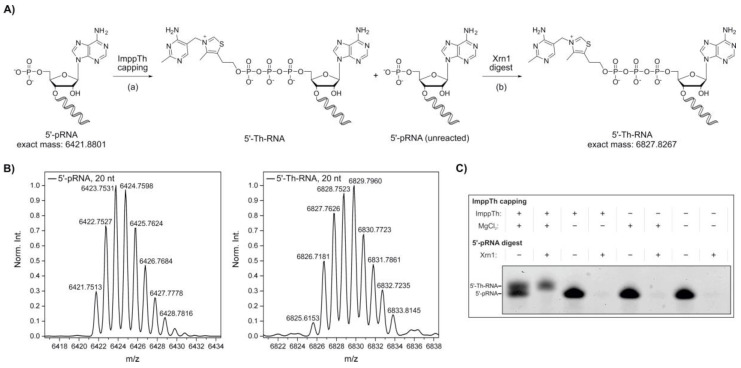
Synthesis of 5′-thiamine-capped RNA by thiamine diphosphate β-*P*‑imidazolide (ImppTh) coupling to 5′-monophosphate RNA (5’-pRNA). (**A**) Schematic illustration of the preparation of 5′-thiamine RNA from 5′-monophosphate RNA (20mer) via (a) ImppTh capping (ImppTh, magnesium chloride, H_2_O, 50 °C) and (b) 5′-monophosphate-dependent exoribonuclease digest using Xrn1, removing unreacted 5′-monophosphate RNA. (**B**) Deconvoluted mass spectra from the high-resolution mass spectrometry (HR-MS, electrospray ionization (ESI), negative mode) analysis of 5′‑monophosphate RNA and 5′-thiamine-capped RNA prepared via ImppTh capping. (**C**) Analysis of varied reaction conditions for ImppTh capping and complete digest of 5′-monophosphate RNA (20mer) from 5′-thiamine-capped RNA by denaturing polyacrylamide gel electrophoresis (see Supplementary Figure S4).

In 2016, our group reported the in vitro synthesis of 5′‑NAD‑capped RNA using imidazolide-activated nicotinamide mononucleotide (ImNMN) [45]. In a similar fashion, we decided to further extend the potential of the ImppTh coupling reaction from 5′‑AMP to 5′‑monophosphate RNA (5′‑pRNA), in an attempt to directly cap RNA sequences with thiamine (Figure 2A). As a substantial amount of RNA I, a small regulatory RNA (sRNA) encoded on the bacterial ColE1 plasmid [47,48], was reported to be NAD-capped in *E. coli* [18], we chose a truncated RNA I 5′‑leader sequence (20 nt, see Supplementary Table S1) as a model system [45].

20mer 5′‑pRNA was treated twice with a thousand-fold excess of ImppTh in aqueous solution at 50 °C (Figure 2A). The reactions were carried out in the presence and absence of 10 mM MgCl_2_. The formation of the desired 5′-thiamine‑capped RNA was confirmed by ESI-MS (Figure 2B). By denaturing polyacrylamide gel electrophoresis (PAGE), a successful capping of the 5′‑pRNA, and therefore slower migration of thiamine-modified RNA species, was only detected for the reaction containing both ImppTh and MgCl_2_ (Figure 2C and Supplementary Figure S4). Furthermore, the presence of divalent magnesium ions did not show any observable RNA hydrolysis effects.

In eukaryotes, uncapped RNA 5′-ends are processed by 5′→3′ exoribonucleases such as the cytoplasmic Xrn1 from *S. cerevisiae*, which specifically degrades 5′‑monophosphate RNA [49,50,51]. Here, Xrn1 was applied after the preparation of 5′‑thiamine RNA with ImppTh to remove all unreacted 5′‑pRNA (Figure 2A). The complete depletion of 5′‑pRNA was monitored by denaturing PAGE, while 5′-thiamine-capped RNA remained untouched by the enzyme (Figure 2B and Supplementary Figure S4). By this method, 5′-thiamine RNA was prepared with yields of approximately 50%. In theory, this preparation is not limited to a certain size of RNA or a specific nucleotide at the 5′‑end apart from it bearing a monophosphate, which can be suggested based on experimental data from our group with 5′-NAD-RNA [45]. 5′-monophosphate RNA can routinely be prepared by polyphosphatase treatment of in vitro transcribed 5′‑triphosphate RNA [52].

**Figure 3:**
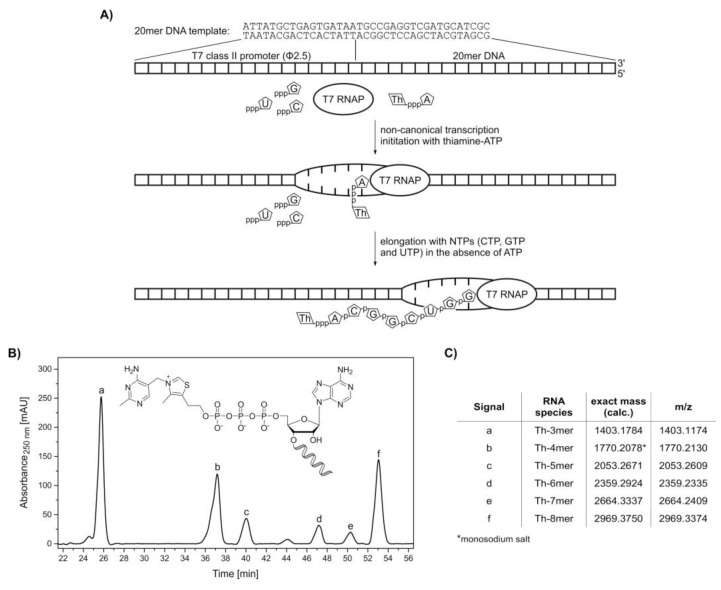
Synthesis of 5′-thiamine-capped RNA (8mer) by in vitro transcription with T7 RNA polymerase using ThATP as a non-canonical initiating nucleotide. (**A**) Schematic illustration of the in vitro transcription of 5′-thiamine-capped RNA with T7 RNA polymerase (T7 RNAP) using thiamine-ATP (ThpppA) and a 20mer DNA template containing a T7 class II promoter (Φ2.5) (Supplementary Table S2). After non-canonical transcription initiation with thiamine-ATP, the elongation process using CTP (pppC), GTP (pppG) and UTP (pppU) in the absence of ATP terminates after passing the nucleotide at the +8 position. In this case, a maximum transcript length of eight nucleotides with the sequence Th‑ACGGCUGG is obtained, which is thiamine-modified at the 5′-end. (**B**) High-performance liquid chromatography (HPLC) analysis of a phenol-ether extracted in vitro transcription reaction with thiamine-ATP in the absence of ATP. (**C**) Assignment of thiamine-capped oligomers to the HPLC peaks via high-resolution mass spectrometry analysis (Supplementary Figure S5).

With the synthesized adenosine-containing dinucleotides thiamine-ATP and thiamine-ADP, in vitro transcription (IVT) experiments with T7 RNA polymerase [53] were conducted in order to determine their potential as NCINs (schematic illustration, see Figure 3A). Besides low unspecific initiation and high RNA yields, the ATP-initiating T7 class II promoter (Φ2.5) also serves as a valuable tool for the incorporation of adenosine derivatives at the 5′-end of RNA sequences [15,54,55,56]. The mechanism of NCIN-mediated transcription initiation has been described in detail for several adenosine-containing coenzymes [16], and it was shown that the concentration of NCINs with respect to nucleoside triphosphates (NTPs), especially ATP, influence the transcription yields of modified RNAs as well as total RNA yields [15].

Transcription initiation with thiamine-ATP was tested in the absence of ATP. In vitro transcription with T7 RNA polymerase was carried out under standard conditions, with two-fold excess of NTPs over thiamine-ATP. The formed oligonucleotide products were monitored by HPLC (Figure 3B). By omission of ATP, the maximum transcript length was eight nucleotides, with thiamine occupying the −1 position. All species ranging from Th-3mer to Th-8mer RNA were confirmed by HR-MS analysis (Figure 3C and Supplementary Figure S5), proving the acceptance of thiamine‑ATP as a non-canonical initiating nucleotide.

The competition of the NCINs thiamine-ATP and thiamine-ADP with ATP for transcription initiation, resulting in a mixture of RNA bearing 5′-thiamine and 5′-triphosphate, was analyzed. In vitro transcriptions with T7 RNA polymerase were carried out under standard conditions, with two-fold excess of thiamine-ATP or thiamine-ADP over NTPs and omission of UTP. The formation of short oligonucleotides was monitored by HPLC (ThATP: Supplementary Figure S6A; ThADP: Supplementary Figure S7A) and their assignment performed by ESI-MS (ThATP: Supplementary Figure S6B–J; ThADP: Supplementary Figure S7B–J). The IVT reactions were repeated in two independent experiments (data not shown).

Peak areas in the HPLC chromatograms (Supplementary Figures S6A and S7A) were calculated and yielded an amount of (55.6% ± 1.7%) and (42.6% ± 3.0%) of ThATP-primed and ThADP-primed 4mer RNA respectively, in comparison to the total amount of canonically and non-canonically primed 4mer RNA species. Therefore, the initiation efficiency with thiamine adenosine dinucleotides, when applied in a two-fold excess over ATP, is approximately equal to canonical initiation for the chosen model system, while thiamine-ATP is more readily incorporated by T7 RNA polymerase than thiamine-ADP.

The approaches we have demonstrated allow for the in vitro preparation of 5′-thiamine RNA, which may be used for the development and evaluation of specific capture techniques that address the 5′-thiamine cap, e.g., via its distinct chemical reactivity. In the identification of natural thiamine-bearing RNA, such a capture step would form the key component of a thiamine-specific capture protocol comparable to the NAD captureSeq [18,19].

Besides the co-enzymatically relevant, carbanionic character of the thiazole C-2 carbon atom [57], the ring opening of the thiazole moiety under alkaline conditions represents a characteristic property of thiamine derivatives. At physiological pH, thiamine is present in its monocationic form. By increasing pH past the pK_a_ of approximately 9.2, the rate-determining nucleophilic addition of one hydroxide anion to the C-2 carbon takes place. A follow-up condensation reaction results in the mentioned opening of the thiazole ring, exposing a formamide-like moiety and a free, reactive thiolate (Supplementary Figure S8) [32,58,59].

**Figure 4:**
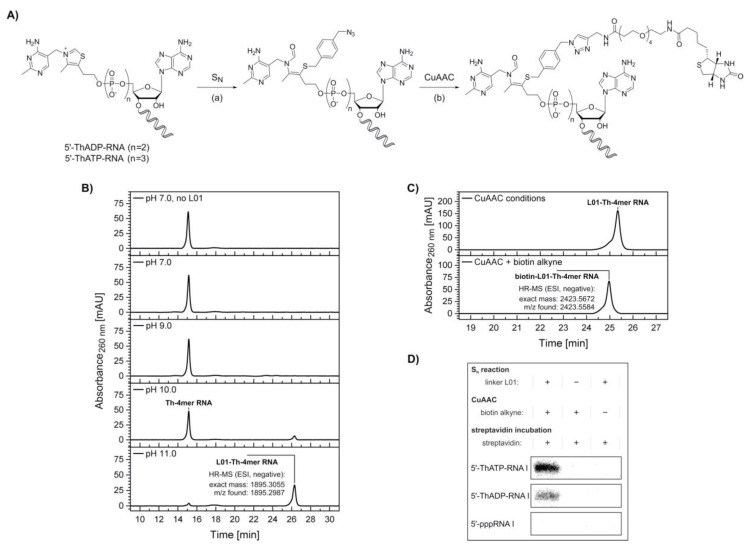
Biotinylation of 5′-thiamine-capped RNA. (**A**) Schematic illustration depicting a combination of thiazole ring opening at elevated pH (see Supplementary Figure S8), (a) nucleophilic substitution (S_N_) at the halogenated benzylic position of the azide-labeled linker L01 molecule (see Supplementary Figure S9) and (b) copper-catalyzed azide-alkyne cycloaddition (CuAAC) with biotin alkyne leading to biotinylation of 5′-thiamine-capped RNA I. (**B**) High-performance liquid chromatography (HPLC) analysis of the nucleophilic substitution reaction with Th-4mer RNA performed at different pH values. High-resolution mass spectrometry (HR-MS) analysis confirmed the formation of the desired azide-labeled Th-RNA species. (**C**) HPLC analysis of the CuAAC reaction with azide-modified 5’-thiamine 4mer RNA (Th-4mer RNA) and biotin alkyne. HR‑MS analysis confirmed the formation of the desired biotinylated Th‑RNA species. (**D**) Analysis of a streptavidin shift assay with [^32^P]-cytidine-labeled 5′-thiamine RNA I (107mer, ThATP‑ and ThADP‑capped) by denaturing polyacrylamide gel electrophoresis. A retardation is visible only for the Th-capped RNA I samples treated via both nucleophilic substitution and CuAAC reaction, whereas no shift is observed for non-fully treated samples or upon similar treatment of control samples of 5′-triphosphate RNA I (see Supplementary Figure S10). Indicators + and − describe the incubation under the respective reaction conditions of nucleophilic substitution or CuAAC in the presence and absence respectively, of linker L01, biotin alkyne and streptavidin.

We decided to utilize this reactivity of thiamine derivatives to design a biochemical tool for the specific modification of in vitro transcribed 5′-thiamine RNA. In a two-step modification protocol, 5′‑thiamine RNA is attached via its thiazole ring-opened form to an electrophilic, azide-modified linker molecule first, before a biotin moiety is introduced via copper-catalyzed azide-alkyne cycloaddition (CuAAC) (Figure 4A).

At the elevated pH necessary for the opening of the thiazole ring, base-catalyzed RNA cleavage by transesterification needs to be considered. This degradation mechanism is promoted by extended reaction times, increasing concentrations of divalent cations and elevated reaction temperatures [60]. For the production of RNA by solid-phase synthesis, however, cleavage from the solid support and deprotection of exocyclic nucleobase amino groups are crucial steps that are routinely performed for up to several hours at temperatures up to 60 °C and a strongly basic pH, e.g., using concentrated aqueous ammonia, while maintaining the integrity of the synthesized RNA strands [61,62,63,64].

To prevent RNA degradation, we designed the nucleophilic substitution with a reactive linker molecule, 1-(azidomethyl)-4-(bromomethyl)benzene (L01) (Supplementary Figure S9), which contains a benzylic bromide, allowing the reaction to proceed within a short time at room temperature (Figure 4A).

To estimate the pH range in which the thiazole ring-opening equilibrium is reasonably shifted towards the reactive thiolate, test reactions were performed with HPLC-purified 5′-thiamine 4mer RNA (Th‑4mer RNA) and the formation of the reaction product with linker L01 monitored by HPLC and confirmed by ESI-MS (Figure 4B). Conversion of the Th-4mer RNA to azide-functionalized L01‑Th‑4mer RNA was confirmed for reaction conditions comprising pH values above pK_a_ 9.2 for the thiazole ring opening, while no significant RNA degradation could be detected. The fractions of L01‑Th‑4mer RNA besides unreacted Th-4mer RNA were calculated as 14% and 91% at pH 10 and pH 11 respectively, with reaction times of 30 min at room temperature. With the azide-modified product, labeling with biotin alkyne was conducted via CuAAC. The clicked product only possessed a slightly changed elution time in HPLC analysis but was confirmed by ESI-MS (Figure 4C), proving the applicability of the reaction sequence for the biotinylation of short transcripts of 5′-thiamine RNA.

In a next step, nucleophilic substitution and CuAAC with biotin alkyne were applied on a [^32^P]‑cytidine-labeled, full-length transcript of RNA I (mixture of 5′-thiamine and 5′-pppRNA) (Figure 4D and Supplementary Figure S10), which was prepared by in vitro transcription in the presence of thiamine-ATP and thiamine-ADP. Under the used conditions, mixtures of 5′-pppRNA I and low amounts of 5′-thiamine-capped RNA I were obtained after PAGE purification and isopropanol precipitation. These mixtures were treated via the reaction sequence of nucleophilic substitution with linker L01 at pH 11 and CuAAC with biotin alkyne and purified via isopropanol precipitation or phenol-ether extraction, respectively. Negative control samples were incubated under the respective reaction conditions in the absence of either linker L01 or biotin alkyne. Interestingly, some degradation tendency was observed in 5′-thiamine RNA I-containing samples incubated at pH 11 in the absence of linker L01 and, thereafter, treated under CuAAC conditions containing copper ions and biotin alkyne, while all other samples showed no comparable degree of degradation (Supplementary Figure S10). In a separate experiment, no significant degree of degradation was monitored for RNA I samples incubated under the reaction conditions of nucleophilic substitution, performed at pH 7 and pH 11, and CuAAC in the presence of linker L01 and biotin alkyne, respectively (Supplementary Figure S11).

Incubation with streptavidin prior to analysis by denaturing PAGE resulted in a retardation of biotin-linked 5′-thiamine RNA I (ThATP- and ThADP-primed) (Figure 4D), whereas the main radioactive species of 5′-pppRNA I contained in the same samples was not shifted (Supplementary Figure S10). Similarly, no retardation was detected for non-fully treated samples or equally treated samples of 5′-pppRNA I (Figure 4D and Supplementary Figure S10), confirming the specific modification of 5′-thiamine RNA in a mixture with 5′-triphosphate RNA.

## 3. Discussion

Adenosine-containing thiamine derivatives have been successfully synthesized by imidazolide-based activation of phosphate groups of the respective thiamine or adenosine species. Thiamine-ADP and the biologically abundant thiamine-ATP were obtained in high yields and successfully purified from minor amounts of side products. With both those dinucleotides and the imidazolide-activated species ImppTh, 5′-thiamine-capped RNA was prepared by in vitro methods.

Despite its inactivation by hydrolysis in aqueous solutions, ImppTh was capable of capping 5′-monophosphate RNA in the presence of divalent magnesium cations. Unreacted 5′-monophosphate RNA was removed by 5′→3′ exonuclease digestion, yielding pure 5′-thiamine RNA in 50% yield. To further increase yields, recovery of unreacted 5′-monophosphate RNA and repeated treatment with ImppTh can be considered. Theoretically, any 5′-triphosphate RNA sequence, independent of length, structure or nucleotide composition, is accessible for thiamine capping by this method, provided it is previously converted to the 5′-monophosphate by, e.g., polyphosphatases.

Furthermore, 5′-thiamine RNA with up to 107 nucleotides, namely the biologically relevant RNA I, was obtained by in vitro transcription with T7 RNA polymerase using thiamine-ATP and thiamine-ADP. The acceptance of thiamine-ATP as a non-canonical initiating nucleotide strongly supports the hypothesis of the existence of thiamine-capped RNA in a variety of organisms. The development of LC-MS-based methods using thiamine-modified model RNAs could lead to the confirmation of this 5′-thiamine cap in total RNA samples, which would confirm a completely new function of thiamine. The lower intracellular concentration of thiamine compared to other NCINs of the B group of vitamins will nonetheless be a major challenge [65].

The formation of a free thiolate by thiazole ring opening was utilized to selectively biotinylate 5′-thiamine RNA next to 5′-triphosphate RNA and for their separation by gel chromatography. The chemical accessibility of the thiamine 5′-cap was thus confirmed, which also makes biochemical modifications, e.g., ribozyme-assisted [66], of 5′-thiamine RNA conceivable. Strategies for metabolic labeling or the specific binding of thiamine-bearing RNA by aptamer structures, such as the Thi-Box riboswitch [67], or thiamine-binding proteins [68] may also be starting points for further research. The presented synthetic methods for in vitro preparation of 5′-thiamine RNA will facilitate and advance the development and evaluation of such specific modifications and capture techniques as well as their implementation into a thiamine-specific capture protocol.

## 4. Materials and Methods

### 4.1. General

Chemicals were purchased from Sigma Aldrich (Steinheim, Germany), Invitrogen (Carlsbad, CA, USA) and Thermo Fisher Scientific (Waltham, MA, USA) and used without further purification. DNA templates, oligonucleotide primers and 5‘‑monophosphorylated RNA were purchased from Integrated DNA Technologies (Coralville, IA, USA). Deionized water was filtered via a MilliQ purification system (Merck Millipore, Burlington, MA, USA). Chemical reactions under argon atmosphere were performed in Schlenk tubes, which were evacuated, heated and flushed with argon three consecutive times. Analysis of chemical reactions was performed by thin-layer chromatography (TLC) using Polygram Sil G/UV254 pre-coated polyester sheets (Macherey Nagel, Düren, Germany) and a UV hand-lamp from Krüss Optronic (Hamburg, Germany). Standard column chromatography was performed on silica gel (60 Å, 40–63 µm; Sigma Aldrich, Steinheim, Germany). For high-performance liquid chromatography (HPLC), setups of the 1100 and 1200 series from Agilent Technologies (Santa Clara, CA, USA) were used with an analytical or semi-preparative HPLC column Luna 5u C18(2) 100 Å, 250 × 4.6 mm and 250 × 15 mm, respectively (Phenomenex, Torrance, CA, USA). Buffered mixtures in water (buffer A: 0.1 M triethylammonium-acetate in water, pH 7.0) and acetonitrile (buffer B: 0.1 M triethylammonium-acetate in acetonitrile:water 4:1, pH 7.0) were utilized as mobile phase for HPLC. HPLC chromatograms were generally recorded at 260 nm and baseline-corrected. For nuclear magnetic resonance (NMR) spectroscopy, substances were dissolved in deuterated solvents and analyzed on a Mercury plus 300 MHz or Mercury plus 500 MHz spectrometer from Varian (Crawley, UK). Chemical shifts were reported in parts per million (ppm) in reference to the deuterated solvent used. Signal multiplicity was abbreviated as s = singulet, d = doublet, t = triplet, q = quartet and m = multiplet. NMR spectra of synthesized compounds are shown in Supplementary Materials (see Supplementary Figures S12–S16). Mass spectrometry (MS) measurements were performed on a micrOTOF QII system (Bruker, Billerica, MA, USA), which was operated in electrospray ionization (ESI) positive or negative mode, with the depiction of the molecular ion as [M]^+^ and [M]^−^, respectively. The calibrant ESI‑L Low-Concentration Tuning Mix (Agilent Technologies, Santa Clara, CA, USA) was applied for high-resolution mass spectrometric (HR‑MS) measurements. Denaturing polyacrylamide gels (8.3 M urea, 0.1 M Tris-borate, 20 mM EDTA, pH 8.3) were prepared using the respective amount of Rotiphorese Sequencing gel concentrate (25%, 19:1; Carl Roth, Karlsruhe, Germany). Denaturing polyacrylamide gel electrophoresis (PAGE) was performed with Tris-borate-EDTA buffer (0.1 M Tris-borate, 20 mM EDTA, pH 8.3). All experiments comprising radioactive samples were performed in a radioactivity control area.

### 4.2. Chemical Synthesis

*((2R,3S,4R,5R)-5-(6-amino-9H-purin-9-yl)-3,4-dihydroxytetrahydrofuran-2-yl)methyl (1H-imidazol-1-yl)phosphonate* (**ImpA**) [41]. A suspension of adenosine 5′‑monophosphate monohydrate (5′‑AMP) (421.4 mg, 1.15 mmol) in dimethylformamide (DMF) (3 mL) was added dropwise to a stirred solution of imidazole (519.9 mg, 7.64 mmol), 2,2′-dithiopyridine (613.0 mg, 2.78 mmol), triphenylphosphine (721.1 mg, 2.75 mmol) and triethylamine (460 µL, 3.30 mmol) in anhydrous DMF (9 mL). Full dissolution of 5′‑AMP was experienced within 10 min. The mixture was stirred at room temperature for 3 h and poured into a cooled solution of sodium perchlorate (143.8 mg, 1.17 mmol) in anhydrous acetone (100 mL). While cooling in a water-ice bath, the product was allowed to precipitate for 1 h. After centrifugation (4000× *g*, 5 min, 15 °C), the pellet was washed with acetone (3 × 10 mL) and diethyl ether (10 mL). After evaporation of residual solvent, the ImpA sodium salt was obtained as a colorless solid (466.2 mg, 96.4%) and stored in an argon-flushed container at −20 °C. ^1^H‑NMR (499.9 MHz, dimethylsulfoxide-*d*_6_ (DMSO‑*d*_6_)): δ [ppm] = 8.39 (s, 1H, C**H**NCNH_2_), 8.13 (s, 1H, C**H**NCCNH_2_), 7.69 (dd, *J* = 1.2 Hz, 1H, NC**H**NP), 7.31 (s, 2H, N**H_2_**), 7.11 (dd, *J* = 1.2 Hz, 1H, C**H**CHNP), 6.87 (dd, *J* = 1.2 Hz, CHC**H**NP), 5.88 (d, *J* = 6.3 Hz, 1H, H‑1′), 4.58 (dd, *J* = 5.4 Hz, 1H, H‑2′), 4.04 (dd, *J* = 4.2, 3.3 Hz, 1H, H‑3′), 3.94 (dt, *J* = 7.2, 3.6 Hz, 1H, H‑4′), 3.81–3.69 (m, 2H, H‑5′). ^13^C‑NMR (75.5 MHz, DMSO‑d_6_): δ [ppm] = 155.9 (1C, **C**NH_2_), 152.7 (1C, **C**HNCNH_2_), 149.7 (1C, **C**CCNH_2_), 135.2 (1C, N**C**HNP), 128.2 (d, 1C, CH**C**HNP), 121.6 (1C, **C**HCHNP), 118.9 (1C, **C**CNH_2_), 86.8 (1C, C‑1′), 83.7 (d, 1C, C‑4′), 73.6 (1C, C‑2′), 70.9 (1C, C‑3′), 64.9 (1C, C‑5′). ^31^P‑NMR (202.3 MHz, DMSO‑d_6_): δ [ppm] = −9.78 (s). HR‑MS (ESI, positive): *m*/*z* calculated for C_13_H_16_N_7_NaO_6_P, 420.0792 [M + H]^+^, 442.0611 [M + Na]^+^; found, 420.0787, 442.0603. MS (ESI, negative): *m*/*z* calculated for C_13_H_16_N_7_NaO_6_P, 396.0827 [M − Na]^−^; found, 396.1.

*3-((4-amino-2-methylpyrimidin-5-yl)methyl)-5-(2-((hydroxy((hydroxy(1H-imidazol-1-yl)phosphoryl)-oxy)phosphoryl)oxy)ethyl)-4-methylthiazol-3-ium* (**ImppTh**). Thiamine pyrophosphate chloride (ThDP) (346.3 mg, 0.75 mmol) was slowly added to a stirred solution of imidazole (349.7 mg, 5.14 mmol), 2,2′-dithiopyridine (414.4 mg, 1.88 mmol), triphenylphosphine (477.4 mg, 1.82 mmol) and triethylamine (300 µL, 2.15 mmol) in anhydrous DMF (8.5 mL). Full dissolution of ThDP was experienced within 2.5 h. The mixture was stirred at room temperature for 3.5 h and poured into a cooled solution of sodium perchlorate (186.2 mg, 1.52 mmol) in anhydrous acetone (85 mL). While cooling in a water-ice bath, the product was allowed to precipitate for 1 h. After centrifugation (4000× *g*, 5 min, 15 °C), the pellet was washed with acetone (3 × 10 mL) and diethyl ether (10 mL). After evaporation of residual solvent, the ImppTh disodium salt was obtained as a pale yellow solid (326.6 mg, 83.7%) and stored in an argon-flushed container at −20 °C. ^1^H‑NMR (499.9 MHz, D_2_O): δ [ppm] = 8.06 (s, 1H, C**H**CCNH_2_), 7.96 (s, 1H, NC**H**NP), 7.32 (dd, *J* = 1.2 Hz, 1H, C**H**CHNP), 7.08 (dd, *J* = 1.2 Hz, 1H, CHC**H**NP), 5.39 (s, 2H, C**H_2_**CCNH_2_), 4.06 (q, *J* = 5.9 Hz, 2H, CH_2_C**H_2_**OP), 3.22 (t, *J* = 5.4 Hz, 2H, C**H_2_**CH_2_OP), 2.55 (s, 3H, C**H_3_**CCS), 2.47 (s, 3H, C**H_3_**CNCNH_2_). ^13^C‑NMR (125.7 MHz, D_2_O): δ [ppm] = 168.9 (1C, CH_3_**C**NCNH_2_), 161.8 (1C, CHC**C**NH_2_), 157.2 (1C, **C**HCCNH_2_), 143.1 (1C, CH_3_**C**CS), 139.6 (1C, N**C**HNP), 135.0 (1C, CH_3_C**C**S), 127.9 (d, 1C, CH**C**HNP), 120.4 (d, 1C, **C**HCHNP), 104.1 (1C, CH**C**CNH_2_), 64.8 (d, 1C, CH_2_**C**H_2_OP), 51.0 (1C, **C**H_2_CCNH_2_), 27.4 (d, 1C, **C**H_2_CH_2_OP), 23.9 (1C, **C**H_3_CNCNH_2_), 11.1 (1C, **C**H_3_CCS). ^31^P‑NMR (202.3 MHz, DMSO‑*d*_6_): δ [ppm] = −11.59 (d, *J* = 22.0 Hz, 1P), −18.89 (d, *J* = 22.0 Hz, 1P). HR‑MS (ESI, positive): *m/z* calculated for C_15_H_21_N_6_O_6_P_2_S^+^, 475.0713 [M]^+^, 497.0532 [M – H + Na]^+^, 519.0330 [M − 2H + 2Na]^+^; found, 475.0706, 497.0520, 519.0352.

*3-((4-amino-2-methylpyrimidin-5-yl)methyl)-5-(2-(((((((((2R,3S,4R,5R)-5-(6-amino-9H-purin-9-yl)-3,4-dihydroxytetrahydrofuran-2-yl)methoxy)(hydroxy)phosphoryl)oxy)(hydroxy)phosphoryl)oxy)(hydroxy)-phosphoryl)oxy)ethyl)-4-methylthiazol-3-ium* (**thiamine‑ATP, ThATP**). Method A: In separate, argon-purged Schlenk tubes, thiamine pyrophosphate chloride (ThDP) (321.5 mg, 0.70 mmol), anhydrous magnesium chloride (161.2 mg, 1.69 mmol) and ImpA (94.2 mg, 0.22 mmol) were dried under reduced pressure for 30 min. Magnesium chloride was suspended in anhydrous DMF (1.2 mL) and added to the ThDP, which was then stirred at room temperature for 1 h before a solution of ImpA in anhydrous DMF (0.8 mL) was added. The mixture was stirred at room temperature for 18 h. Then, the solvent was fully evaporated under reduced pressure while stirring at room temperature. Residual solid was dissolved in 0.1 M triethylammonium-acetate buffer (pH 7.0) and purified by HPLC (5–8% buffer B in 50 min, 5.0 mL/min), yielding the product as a pale-yellow solid. Method B: In separate, argon-purged Schlenk tubes, adenosine 5′‑monophosphate monohydrate (5′-AMP) (700.4 mg, 1.92 mmol), anhydrous magnesium chloride (274.3 mg, 2.88 mmol) and ImppTh (198.8 mg, 0.38 mmol) were dried under reduced pressure for 1 h. Magnesium chloride was suspended in anhydrous DMF (1.8 mL) and added to the 5′‑AMP, which was then stirred at room temperature for 1 h before a solution of ImppTh in anhydrous DMF (1.2 mL) was added. The mixture was stirred at room temperature for 18 h. Then, the solvent was fully evaporated under reduced pressure while stirring at room temperature. Residual solid was dissolved in 0.1 M triethylammonium-acetate buffer (pH 7.0) and purified by HPLC (6–9% buffer B in 50 min, 6.0 mL/min), yielding the product as a pale-yellow solid. ^1^H‑NMR (499.9 MHz, D_2_O): δ [ppm] = 9.41 (s, 1H, SC**H**N), 8.49 (s, 1H, C**H**NCCNH_2_), 8.21 (s, 1H, C**H**NCNH_2_), 7.92 (s, 1H, C**H**CCNH_2_), 6.09 (s, *J* = 6.0 Hz, 1H, H‑1′), 5.36 (s, 2H, C**H_2_**CCNH_2_), 4.76 (dd, *J* = 5.6 Hz, 1H, H‑2′), 4.54–4.51 (m, 1H, H‑3′), 4.39–4.36 (m, 1H, H‑4′), 4.23–4.20 (m, 2H, H‑5′), 4.20–4.15 (m, 2H, CH_2_C**H_2_**OP), 3.26–3.22 (m, 2H, C**H_2_**CH_2_OP), 2.52 (s, 3H, C**H_3_**CCS), 2.47 (s, 3H, C**H_3_**CNCNH_2_). ^13^C‑NMR (125.7 MHz, D_2_O): δ [ppm] = 165.4 (1C, CH_3_**C**NCNH_2_), 162.0 (1C, CHC**C**NH_2_), 155.1 (1C, CHN**C**NH_2_), 152.4 (d, 1C, **C**HNCNH_2_), 150.3 (d, 1C, **C**HCCNH_2_), 148.9 (1C, C**C**CNH_2_), 143.2 (1C, CH_3_**C**CS), 139.9 (1C, **C**HNCCNH_2_), 135.4 (d, 1C, CH_3_C**C**S), 118.3 (1C, CC**C**NH_2_), 105.1 (1C, CH**C**CNH_2_), 86.6 (1C, C‑1′), 83.9 (d, 1C, C‑4′), 74.1 (1C, C‑2′), 70.3 (1C, C‑3′), 65.2 (d, 1C, C‑5′), 64.8 (d, 1C, CH_2_**C**H_2_OP), 50.3 (1C, **C**H_2_CCNH_2_), 27.5 (1C, **C**H_2_CH_2_OP), 22.1 (1C, **C**H_3_CNCNH_2_), 11.1 (1C, **C**H_3_CCS). ^31^P‑NMR (202.3 MHz, D_2_O): δ [ppm] = −10.99 (d, *J* = 19.5 Hz, 1P), −11.23 (d, *J* = 19.5 Hz, 1P), −22.88 (t, *J* = 19.2 Hz, 1P). HR‑MS (ESI, negative): *m/z* calculated for C_22_H_30_N_9_O_13_P_3_S, 752.0824 [M − H]^−^; found, 752.0818 (method A), 752.0839 (method B).

*3-((4-amino-2-methylpyrimidin-5-yl)methyl)-5-(2-(((((((2R,3S,4R,5R)-5-(6-amino-9H-purin-9-yl)-3,4-dihydroxytetrahydrofuran-2-yl)methoxy)(hydroxy)phosphoryl)oxy)(hydroxy)phosphoryl)oxy)ethyl)-4-methylthiazol-3-ium* (**thiamine‑ADP, ThADP**). In separate, argon-purged Schlenk tubes, thiamine monophosphate chloride dihydrate (ThMP) (98.9 mg, 0.24 mmol), anhydrous magnesium chloride (164.4 mg, 1.73 mmol) and ImpA (70.0 mg, 0.17 mmol) were dried under reduced pressure for 30 min. Magnesium chloride was suspended in anhydrous DMF (1.2 mL) and added to the ThMP, which was then stirred at room temperature for 1 h before a solution of ImpA in anhydrous DMF (0.8 mL) was added. The mixture was stirred at room temperature for 18 h. Then, the solvent was fully evaporated under reduced pressure while stirring at room temperature. Residual solid was dissolved in 0.1 M triethylammonium-acetate buffer (pH 7.0) and purified by HPLC (5‑8% buffer B in 50 min, 5.0 mL/min), yielding the product as a pale-yellow solid. HR‑MS (ESI, negative): *m/z* calculated for C_22_H_29_N_9_O_10_P_2_S, 672.1161 [M − H]^−^; found, 672.1169.

*1-(azidomethyl)-4-(bromomethyl)benzene* (**L01**) [69]. A solution of sodium azide (61.6 mg, 0.95 mmol) in anhydrous DMF (5 mL) was added to a solution of α,α’-dibromo-*p*-xylene (250.0 mg, 0.95 mmol) in dry DMF (10 mL). Under light exclusion, the reaction was stirred at room temperature for 44 h. Reaction control was performed by normal phase (NP)‑TLC (cyclohexane (C_6_H_6_), R_f_ = 0.11 (L01), R_f_ = 0.21 (α,α’-dibromo-*p*-xylene)). The solvent was evaporated under reduced pressure. Residual solid was extracted three times with dichloromethane (DCM) (4 mL each). The organic phases were pooled, dried over anhydrous MgSO_4_, filtered and concentrated under reduced pressure. The crude product was purified by silica column chromatography (C_6_H_6_, then C_6_H_6_/DCM 1:1). Solvent was evaporated under reduced pressure yielding the product as a pale-yellow, viscous liquid (101.3 mg, 47.3%). ^1^H‑NMR (300.0 MHz, CDCl_3_): δ [ppm] = 7.33 (d, *J* = 8.1 Hz, 2H, C**H**CCH_2_Br), 7.21 (d, *J* = 8.1 Hz, 2H, C**H**CCH_2_Br), 4.41 (s, 2H, C**H_2_**Br), 4.26 (s, 2H, C**H_2_**N_3_). ^13^C‑NMR (75.4 MHz, CDCl_3_): δ [ppm] = 138.0 (1C, **C**CH_2_Br), 135.8 (1C, **C**CH_2_N_3_), 129.6 (2C, **C**HCCH_2_Br), 128.7 (2C, **C**HCCH_2_N_3_), 54.5 (1C, **C**H_2_N_3_), 33.0 (1C, **C**H_2_Br).

### 4.3. Preparation, Purification, Modification and Analysis of Ribonucleic Acids

#### 4.3.1. Preparation of 5′-Thiamine-Capped RNA via ImppTh Reaction and Xrn1 Digest

5′-Monophosphate RNA (20 nt, see Supplementary Table S1) (10 µM, 0.5 nmol) was incubated in the presence of 10 mM ImppTh and 10 mM MgCl_2_ at 50 °C for 1 h. Then, a second addition of ImppTh was performed, increasing the concentration to 20 mM, followed by further incubation at 50 °C for 1 h. Modified and unmodified RNA was purified via ethanol precipitation and 0.2 nmol of RNA were further incubated in the presence of 1 U Xrn1 (New England BioLabs Inc., Ipswich, MA, USA) in 1 X NEB buffer 3 (New England BioLabs Inc., Ipswich, MA, USA) at 37 °C for 2 h. RNA was purified from Xrn1 by phenol-chloroform and ether extraction, followed by ethanol precipitation. Samples of the reaction with ImppTh and Xrn1 digest were analyzed by 20% denaturing PAGE and RNA bands visualized on a Typhoon FLA 9500 biomolecular imager (GE Healthcare, Chicago, IL, USA) upon staining with SYBR Gold (Invitrogen, Carlsbad, CA, USA). ESI-MS analysis was conducted to validate the identity of 5′-thiamine RNA.

#### 4.3.2. Preparation of DNA Templates for In Vitro Transcription

The DNA template for RNA I was PCR amplified, while other DNA templates were annealed by incubation of complementary oligonucleotide primers (see Supplementary Table S2).

#### 4.3.3. In Vitro Transcription

In vitro transcriptions were performed in the presence of 5 µM DNA template, 4 mM NCIN (ThATP, ThADP or 3′-dephospho CoA), 2 mM ATP, 2 mM CTP, 2 mM GTP, 2 mM UTP, 40 mM Tris-HCl (pH 8.1), 1 mM spermidine, 22 mM MgCl_2_, 0.01% Triton-X-100, 10 mM DTT, 5% DMSO and 0.1 µg/µL T7 RNA polymerase (lab stock, 1 mg/mL) and incubated at 37 °C for 3.5 h. Radioactively labeled RNA was prepared accordingly in the presence of 1 µCi/µL [α‑^32^P]‑CTP. RNA was purified by phenol-chloroform and ether extraction, followed by semi-preparative HPLC (<20 nt) or denaturing PAGE and isopropanol precipitation (≥20 nt).

#### 4.3.4. Modification of the 5′-Thiamine Cap via Nucleophilic Substitution and Copper-Catalyzed Azide-Alkyne Cycloaddition

The nucleophilic substitution using 5′‑thiamine derivatives was performed in the presence of 10 µM thiamine-ATP or 4mer RNA (5′‑ThATP, 5′-ThADP and 5′‑triphosphate), 200 µM linker L01, 50 mM buffer (Tris-HCl at pH 7.0, 9.0 and CAPS at pH 10.0, 11.0) and 2.0% *v*/*v* DMSO and incubated at 25 °C for 1 h. The copper-catalyzed azide-alkyne cycloaddition was performed in the presence of 25 µM azide-modified thiamine-ATP or 4mer RNA, 500 µM biotin alkyne (PEG_4_ carboxamide-propargyl biotin), 100 µM CuSO_4_, 500 µM Tris(3-hydroxypropyl-triazolylmethyl)amine (THPTA ligand), 50 mM 4-(2-hydroxyethyl)-1-piperazineethanesulfonic acid (HEPES) (pH 7.5), 200 mM NaCl, 200 mM KCl and 2.5% *v*/*v* DMSO and incubated at 25 °C for 30 min. Both reactions were analyzed via HPLC, followed by ESI-MS measurements.

#### 4.3.5. Streptavidin Retardation Assay of 5′-Thiamine-Capped RNA

Radioactively labeled RNA I (5′-ThpppA, 5′-ThppA, 5′-CoA and 5′-ppp) was incubated in the presence of 4 µM 1-(azidomethyl)-4-(bromomethyl)benzene (L01), 50 mM CAPS (pH 11.0) and 0.4% *v*/*v* DMSO at 25 °C for 1 h. RNA was purified via isopropanol precipitation. For copper-catalyzed azide-alkyne cycloaddition (CuAAC), the redissolved RNA was incubated in the presence of 500 µM biotin alkyne, 100 µM CuSO_4_, 500 µM THPTA ligand, 1 mM sodium ascorbate, 50 mM HEPES (pH 7.5), 200 mM NaCl, 200 mM KCl and 2.5% *v*/*v* DMSO at 25 °C for 30 min. RNA was purified by phenol-chloroform and ether extraction and the aqueous solvent was removed to dryness under reduced pressure. RNA was redissolved, incubated in the presence of 0.4 µg/µL streptavidin, 25 mM HEPES (pH 7.5), 100 µM NaCl and 100 µM KCl at 25 °C for 5 min and analyzed by 10% denaturing polyacrylamide gel electrophoresis. Radioactive RNA bands were visualized on a Typhoon FLA 9500 biomolecular imager (GE Healthcare, Chicago, IL, USA) using storage phosphor screens (GE Healthcare, Chicago, IL, USA).

## Supplementary Materials

The following are available online, Figure S1: HPLC purification of thiamine-ATP and thiamine-ADP, Figure S2: ImppTh degradation in aqueous solution, Figure S3: synthesis and purification of thiamine-ADP, Figure S4: thiamine-capping of 5′-monophosphate RNA (20mer), Figure S5: HR-MS analysis of in vitro transcribed 5′-thiamine RNA (8mer), Figure S6: in vitro transcription of 5′-thiamine RNA (4mer) with ThATP and ATP, Figure S7: in vitro transcription of 5′-thiamine RNA (4mer) with ThADP and ATP, Figure S8: thiazole ring-opening equilibrium of thiamine derivatives, Figure S9: nucleophilic substitution with linker L01, Figure S10: streptavidin shift assay with radioactively labeled RNA I, Figure S11: Analysis of RNA integrity in S_N_ and CuAAC conditions, Figures S12–S16: NMR spectra, Table S1: RNA sequences, Table S2: oligonucleotide primers and DNA sequences.
