# Supplementary material for: Synthesis of 5′-Thiamine-Capped RNA
Source: Molecules. 2020 Nov 24;25(23):5492. doi: 10.3390/molecules25235492 (PMC7727699; doi:10.3390/molecules25235492)
Supplement: Supplementary file 1 [file molecules-25-05492-s001.pdf]

Supplementary Materials

Synthesis of 5′-Thiamine-Capped RNA

Marvin Möhler, Katharina Höfer and Andres Jäschke*

Institute of Pharmacy and Molecular Biotechnology, Heidelberg University, Im Neuenheimer Feld 364, 69120 Heidelberg, Germany

* Correspondence: jaeschke@uni-hd.de

Contents

Figure S1. HPLC purification of thiamine-ATP and thiamine-ADP 2
Figure S2. ImppTh degradation in aqueous solution 3
Figure S3. Synthesis and purification of thiamine-ADP 4
Figure S4. Thiamine-capping of 5′-monophosphate RNA (20mer) 5
Figure S5. HR-MS analysis of in vitro transcribed 5′-thiamine RNA (8mer) 6
Figure S6. In vitro transcription of 5′-thiamine RNA (4mer) with ThATP and ATP 7
Figure S7. In vitro transcription of 5′-thiamine RNA (4mer) with ThADP and ATP.... 8
Figure S8. Thiazole ring-opening equilibrium of thiamine derivatives 9
Figure S9. Nucleophilic substitution with linker L01 9
Figure S10. Streptavidin shift assay with radioactively labeled RNA I 10
Figure S11. Analysis of RNA integrity in S_N and CuAAC conditions 11
Table S1. RNA sequences. 12
Table S2. Oligonucleotide primers and DNA templates 12
Figure S12–16. NMR spectra 13–17

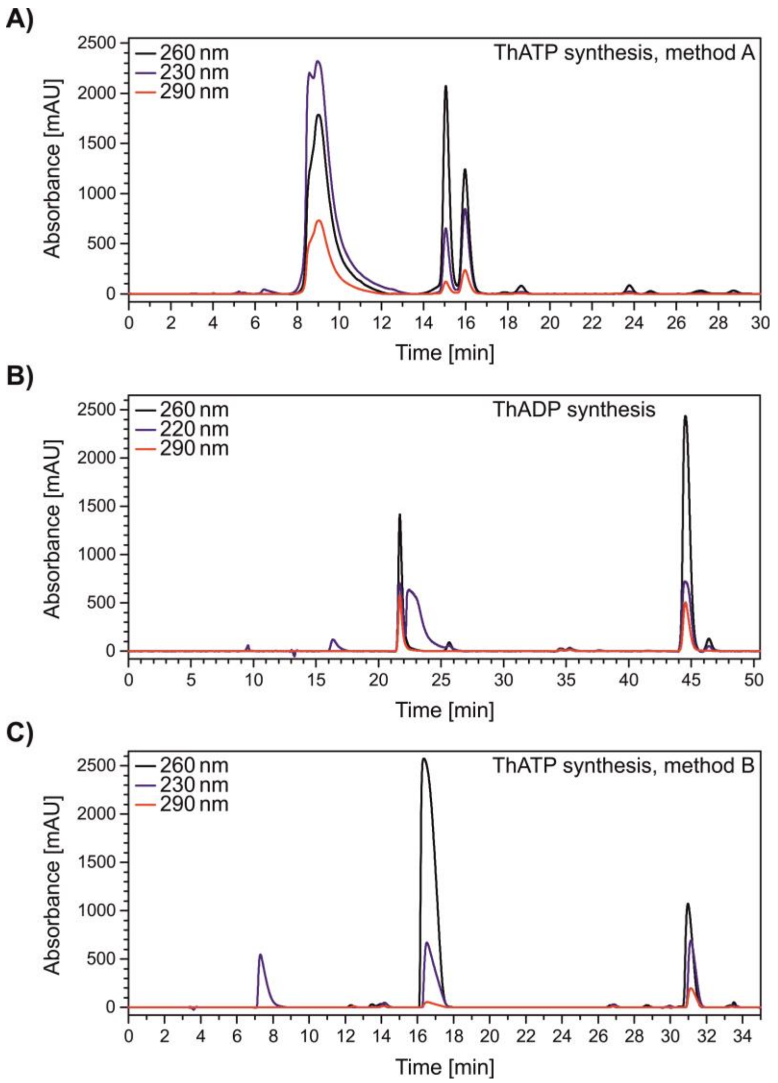

Figure S1. HPLC purification of thiamine-ATP and thiamine-ADP, full chromatograms. (A) Chromatograms (recorded at 230 nm (blue), 260 nm (black) and 290 nm (red)) for the HPLC purification of ThATP prepared by ThATP synthesis method A. Major peaks were assigned to ThDP and 5′-AMP (both 8–12 min), AppA (15 min) and ThATP (16 min) by ESI-MS analysis. Method: 6–9% buffer B in 30 min, 6.0 mL/min, semi-preparative HPLC column. (B) Chromatograms (recorded at 220 nm (blue), 260 nm (black) and 290 nm (red)) for the HPLC purification of ThADP. Major peaks were assigned to ThMP (22 min), ThATP (44.5 min) and AppA (46.5 min) by ESI-MS analysis. Method: 5–8% buffer B in 50 min, 5.0 mL/min, semi-preparative HPLC column. (C) Chromatograms (recorded at 230 nm (blue), 260 nm (black) and 290 nm (red)) for the HPLC purification of ThATP prepared by ThATP synthesis method B. Major peaks were assigned to 5′-AMP (17 min) and ThATP (31 min) by ESI-MS analysis. Method: 1–5% buffer B (25 min) and 5–15% buffer B (25–35 min), 1.0 mL/min, analytical HPLC column.

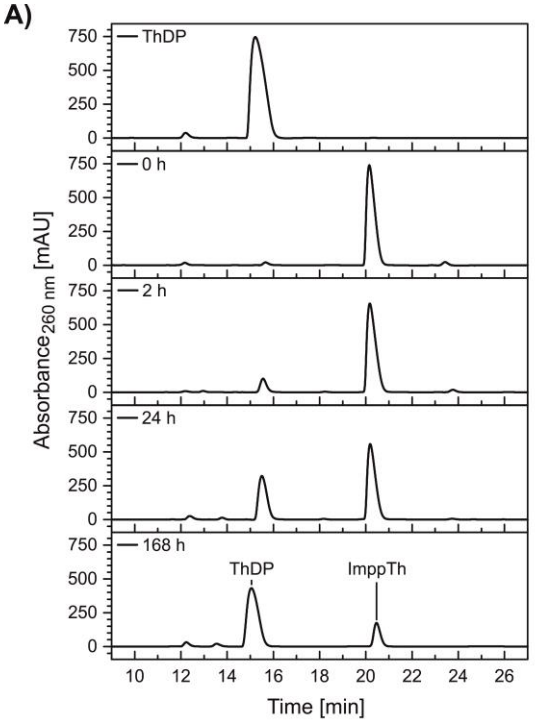

B)

Incubation time	% ImppTh
0 h*	98.0%
2 h	90.0%
24 h	62.7%
7 d	19.0%

*time-point 0 h: ImppTh dissolution in buffer A and injection into the HPLC system

Figure S2. ImppTh degradation to ThDP in buffered, aqueous solution. (A) HPLC analysis of ImppTh degradation in buffered solution (buffer A: 0.1 M triethylammonium-acetate in water, pH 7.0) after incubation for 0 h, 2 h, 24 h and 168 h. The hydrolysis product was confirmed to be ThDP using a control sample (top) as well as ESI-MS analysis. (B) Table depicting the remaining amount of ImppTh in solution, calculated from the peak area for ImppTh and ThDP extracted from the baseline-corrected HPLC chromatograms.

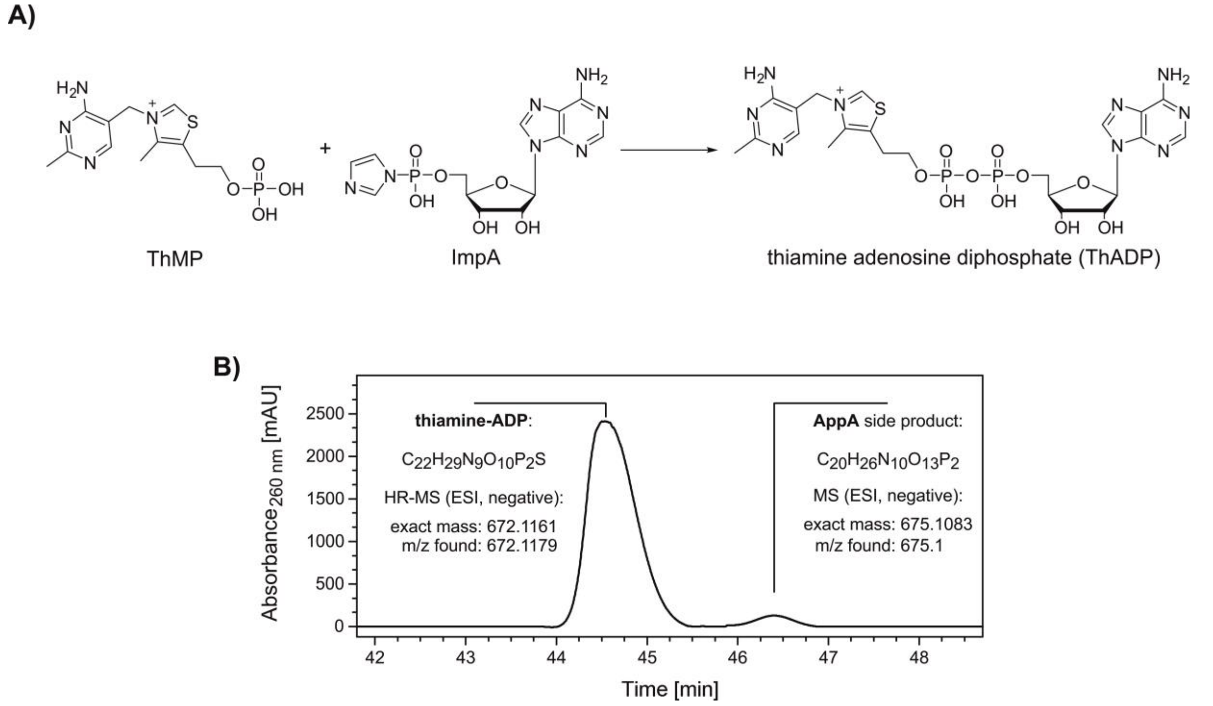

Figure S3. Synthesis and purification of thiamine-ADP. (A) Synthesis scheme of ThADP via coupling of the imidazolide-activated species ImpA to ThMP; 1. ThMP, $MgCl_2$, DMF, rt, 2. ImpA; (B) HPLC and HR-MS analysis confirm the formation of ThADP. By homodimerization, the side product AppA is formed.

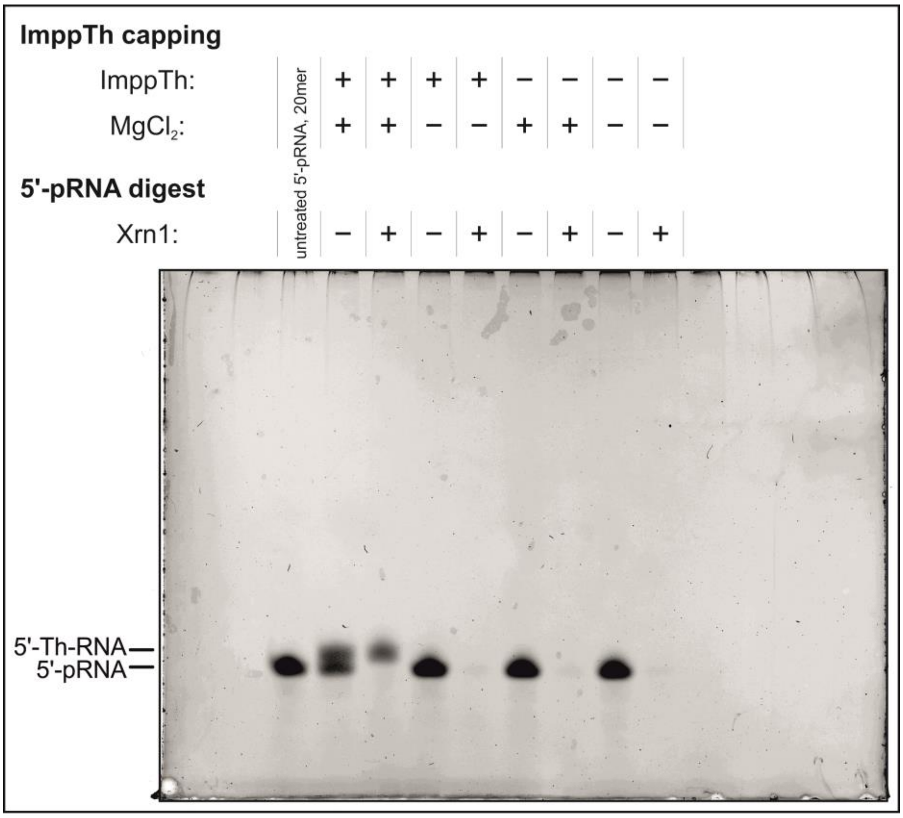

Figure S4. Analysis of ImppTh capping of 5′-monophosphate RNA (20mer) and complete Xrn1 digest of unreacted RNA from 5′-thiamine-capped RNA by denaturing 20% polyacrylamide gel electrophoresis (SYBR Gold stain).

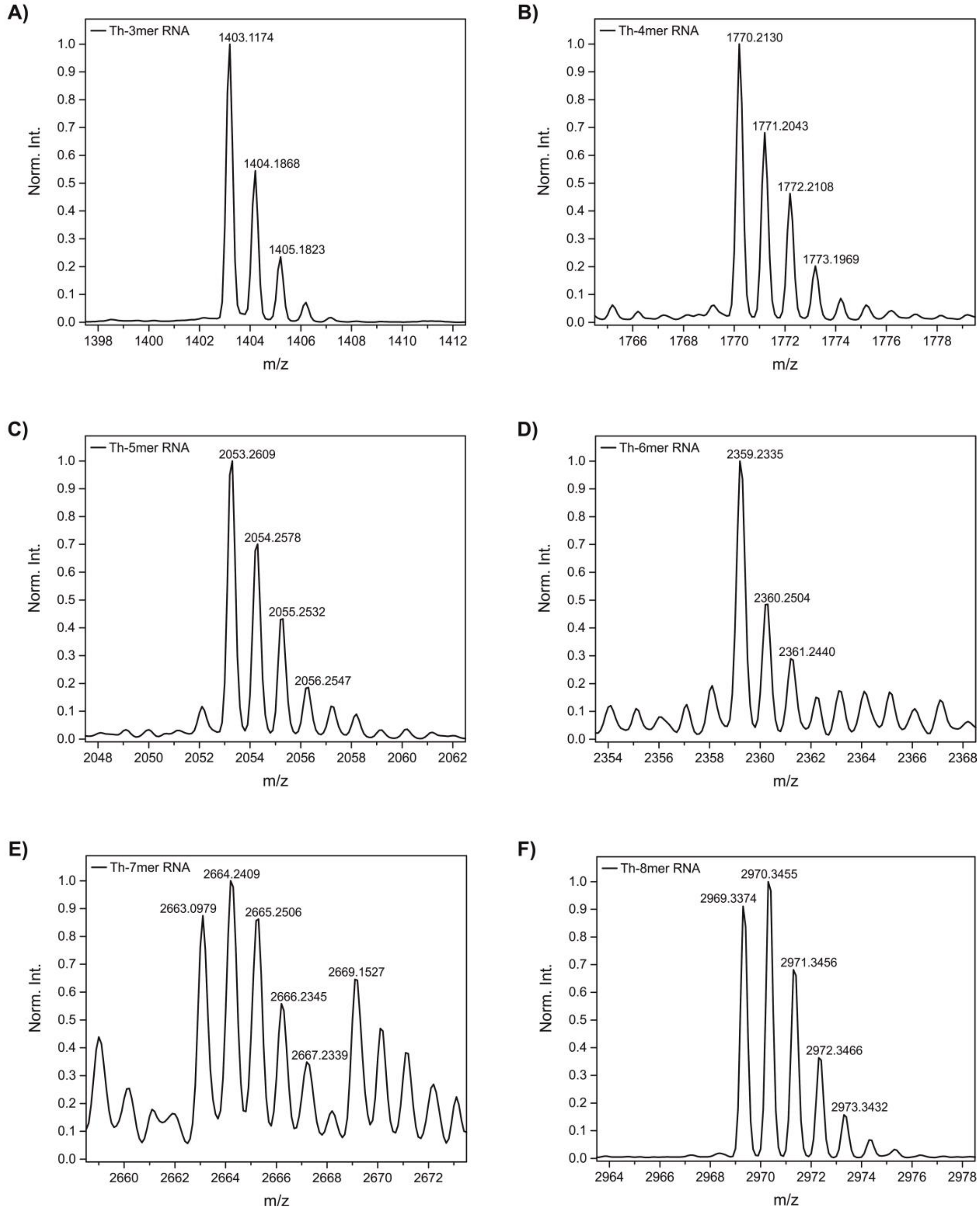

Figure S5. Deconvoluted mass spectra of thiamine-capped oligomers prepared via in vitro transcription using T7 RNA polymerase. (A) Th-3mer RNA (calc. 1403.1784, found 1403.1174). (B) Th-4mer RNA (as monosodium salt, calc. 1770.2078, found 1770.2130). (C) Th-5mer RNA (calc. 2053.2671, found 2053.2609). (D) Th-6mer RNA (calc. 2359.2924, found 2359.2335). (E) Th-7mer RNA (calc. 2664.3337, found 2664.2409). (F) Th-8mer RNA (calc. 2969.3750, found 2969.3374).

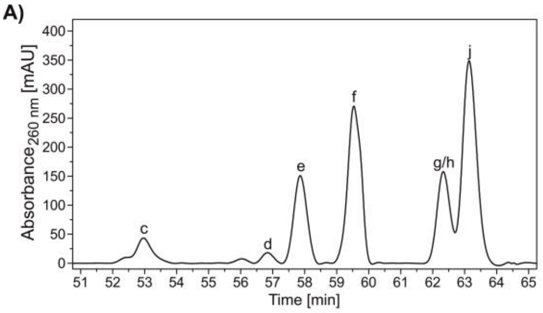

B)

Signal	RNA species	exact mass (calc.)	m/z
c	3mer pppRNA	1141.0896	1141.0839
d	4mer pRNA	1326.2043	1326.2097
e	Th-3mer RNA	1387.1835	1387.1756
f	4mer pppRNA	1486.1370	1486.1503
g	5mer pppRNA*	1791.1783	1791.1729
h	Th-5mer RNA*	2037.2772	2037.2596
j	Th-4mer RNA	1732.2309	1732.2264

*5mer RNA with 5'-triphosphate and 5'-thiamine-triphosphate eluted at the same time

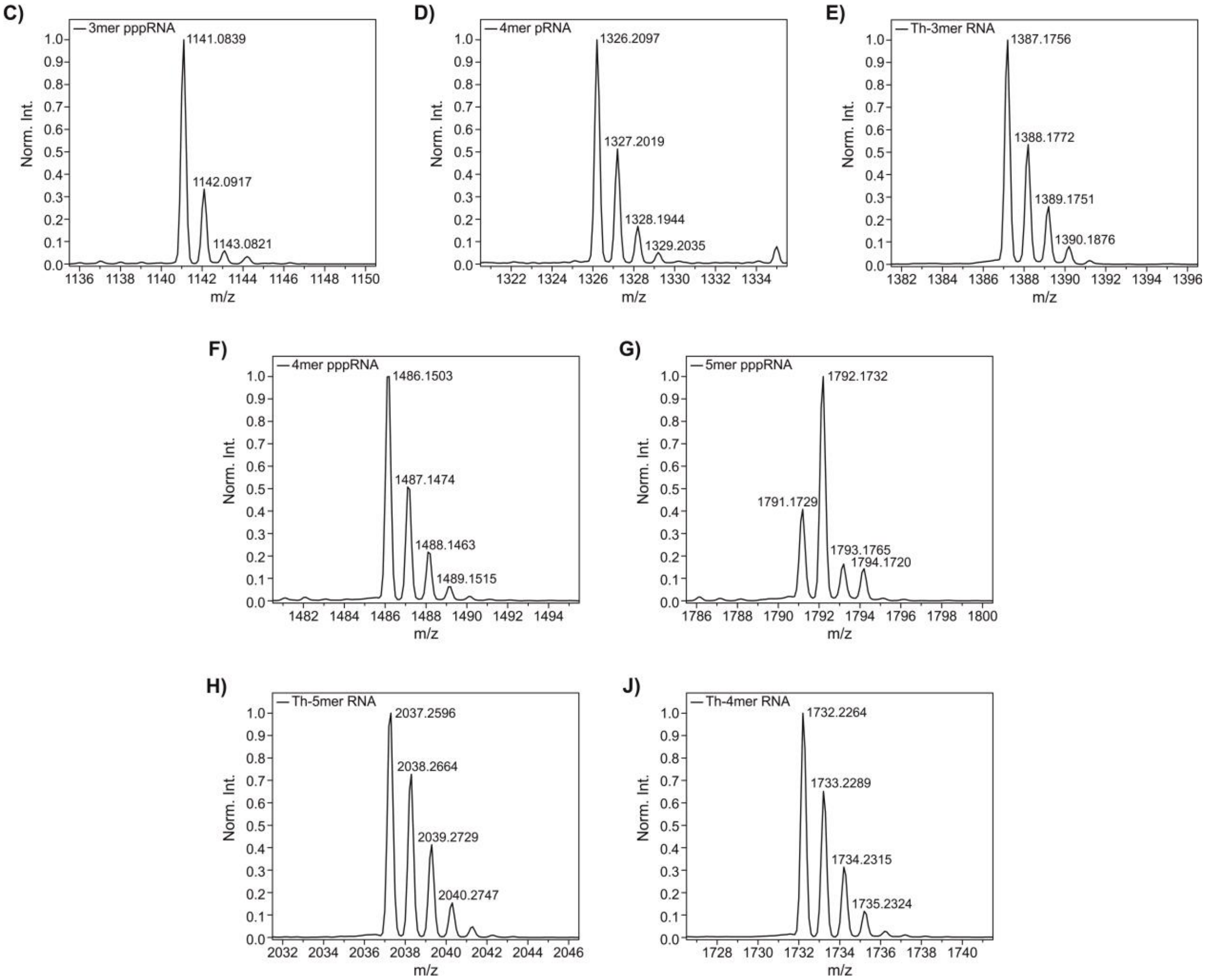

Figure S6. Synthesis of 5′-thiamine-capped RNA by in vitro transcription with T7 RNA polymerase using thiamine-ATP (ThATP) as a non-canonical initiating nucleotide in the presence of ATP. (A) HPLC analysis of phenol-ether extracted IVT reactions. (B) Assignment of thiamine-capped oligomers to the HPLC peaks via HR-MS analysis. Deconvoluted mass spectra of RNA oligomers (C–J) confirm the incorporation of ThATP.

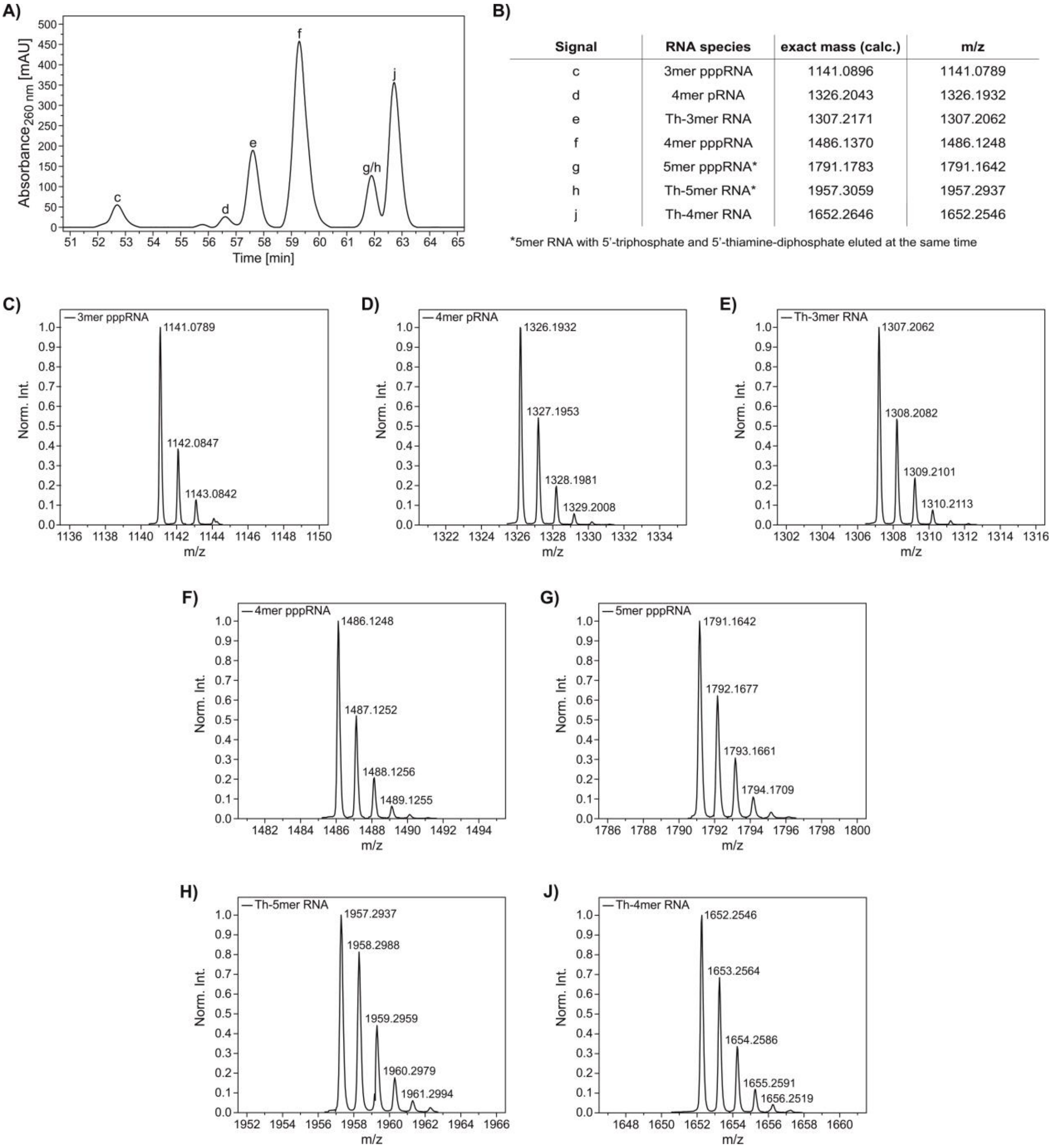

Signal	RNA species	exact mass (calc.)	m/z
c	3mer pppRNA	1141.0896	1141.0789
d	4mer pRNA	1326.2043	1326.1932
e	Th-3mer RNA	1307.2171	1307.2062
f	4mer pppRNA	1486.1370	1486.1248
g	5mer pppRNA*	1791.1783	1791.1642
h	Th-5mer RNA*	1957.3059	1957.2937
j	Th-4mer RNA	1652.2646	1652.2546

*5mer RNA with 5'-triphosphate and 5'-thiamine-diphosphate eluted at the same time

Figure S7. Synthesis of 5′-thiamine-capped RNA by in vitro transcription with T7 RNA polymerase using thiamine-ADP (ThADP) as a non-canonical initiating nucleotide in the presence of ATP. (A) HPLC analysis of phenol-ether extracted IVT reactions. (B) Assignment of thiamine-capped oligomers to the HPLC peaks via HR-MS analysis. Deconvoluted mass spectra of RNA oligomers (C–J) confirm the incorporation of ThADP.

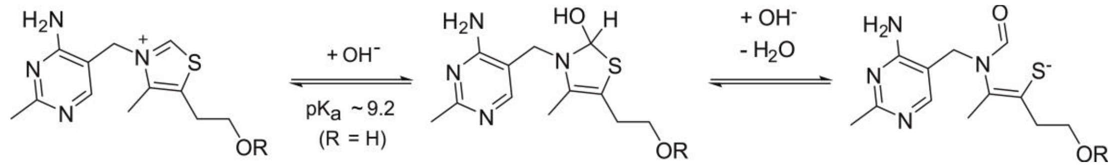

Figure S8. Schematic illustration of the thiazole ring-opening equilibrium of thiamine derivatives at elevated pH. The first addition of a hydroxide anion, with a pK_a of approximately 9.2 for ThOH, is the rate-determining step, which is followed by the thiazole ring opening exposing a reactive thiolate[1].

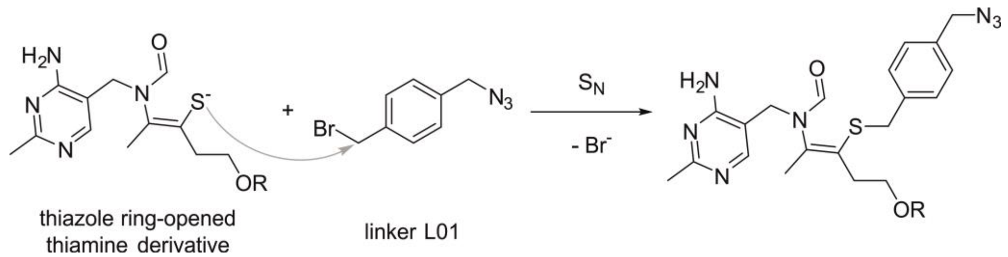

Figure S9. Schematic illustration of the nucleophilic substitution reaction of the thiolate of a thiazole ring-opened thiamine derivative (see Figure S8) with the reactive linker molecule L01 (1-(azidomethyl)-4-(bromomethyl)benzene).

[1] Pérez-Caballero, G.; Pérez-Arévalo, J.F.; Morales-Hipólito, E.A.; Carbajal-Arenas, M.E.; Rojas-Hernández, A. Potentiometric study of acid-base properties of thiamine hydrochloride and thiamine mononitrate in aqueous medium. *J. Mex. Chem. Soc.* **2011**, *55*, 126-131.

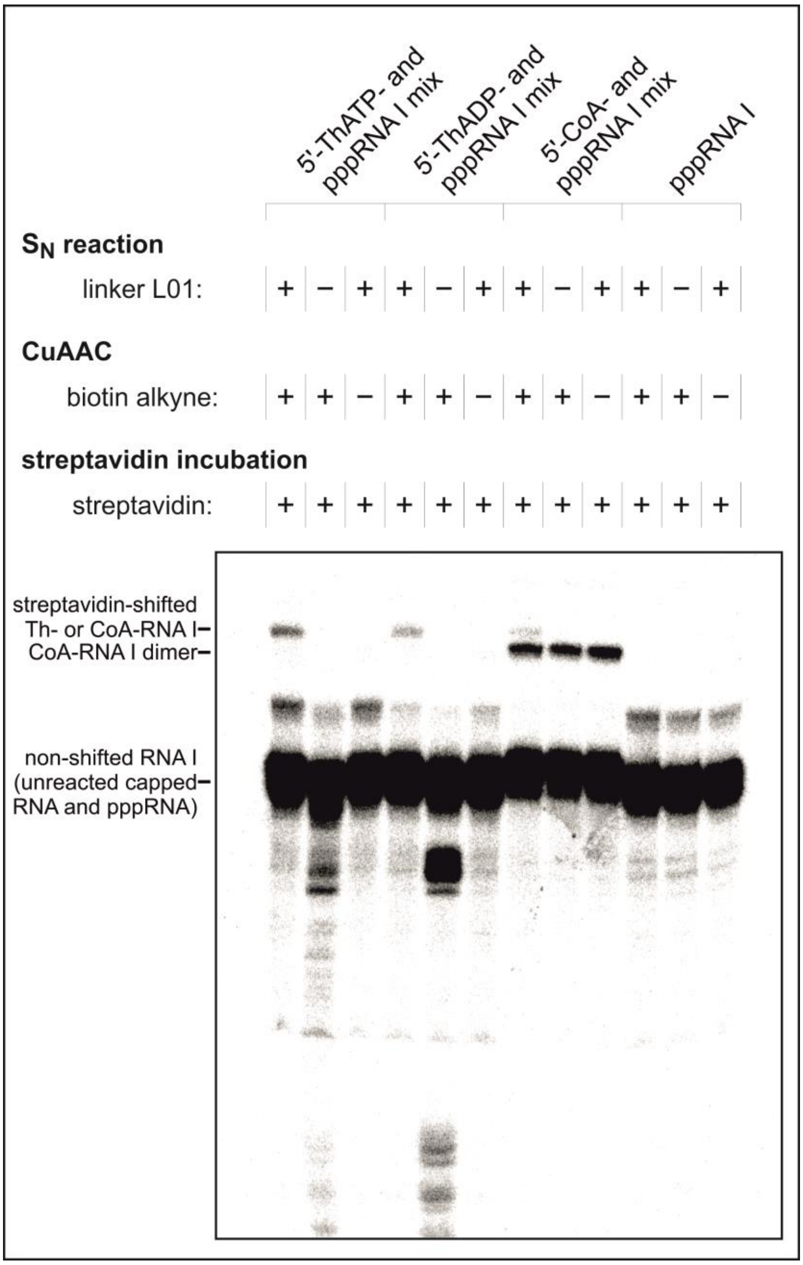

Figure S10. Streptavidin shift assay with radioactively labeled RNA I (107mer, ThATP-, ThADP-, dephospho-CoA-capped RNA I applied in a mixture with 5′-pppRNA I). Analysis by denaturing 10% polyacrylamide gel electrophoresis. Indicator + and − describe the incubation under the respective reaction conditions of nucleophilic substitution or CuAAC in the presence and absence, respectively, of linker L01, biotin alkyne and streptavidin.

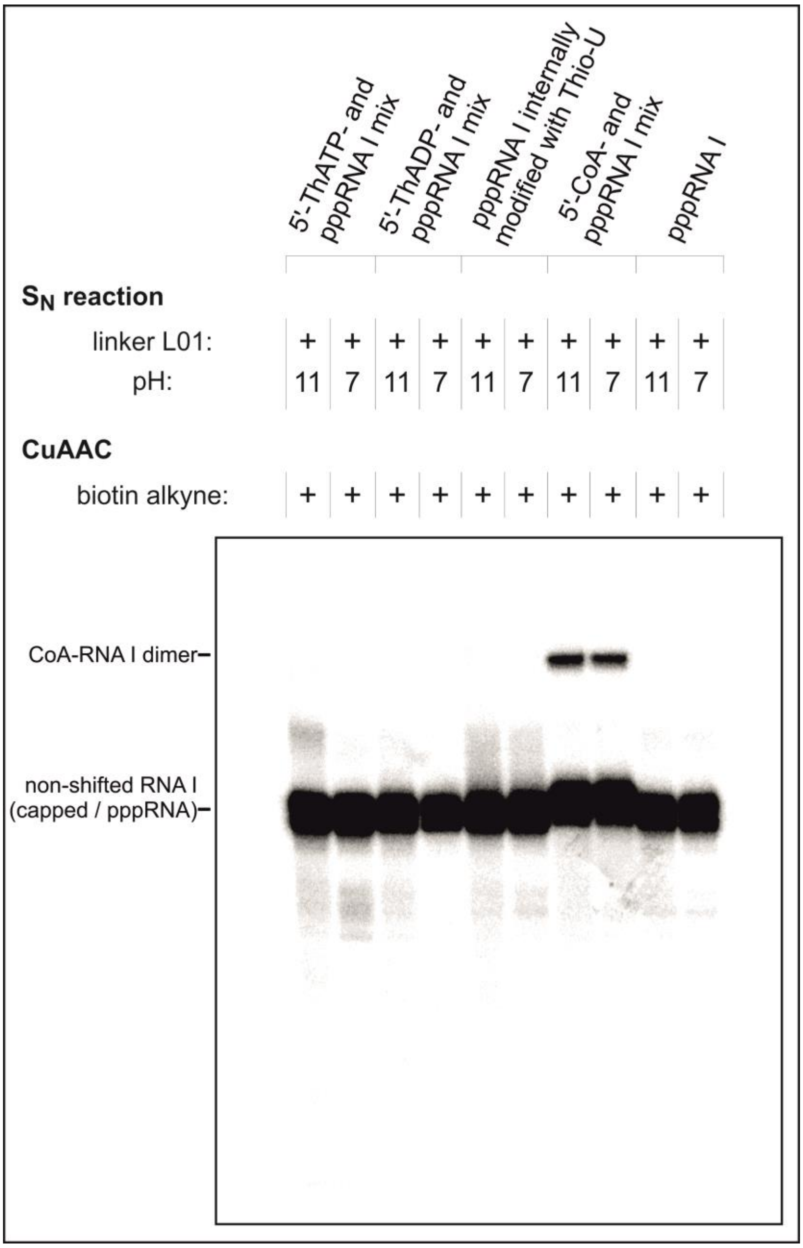

Figure S11. Analysis of RNA integrity in nucleophilic substitution (S_N) and CuAAC conditions, performed with radioactively labeled RNA I (107mer, ThATP-, ThADP-, dephospho-CoA-capped RNA I applied in a mixture with 5′-pppRNA I and internally thiouridine (thio-U)-modified 5′-pppRNA I). Analysis by denaturing 10% polyacrylamide gel electrophoresis. Indicator + describes the incubation under the respective reaction conditions of nucleophilic substitution (performed either at pH 11.0, 50 mM CAPS buffer, or at pH 7.0, 50 mM Tris-HCl buffer) or CuAAC in the presence of linker L01 or biotin alkyne.

Table S1. RNA sequences used in this study.

RNA	Sequence (5′–3′)
4mer RNA	ppp-ACAG
4mer 5′-Th-RNA	Th-ppp-ACAG
8mer 5′-Th-RNA	Th-ppp-ACGGCTCC
20mer 5′-pRNA	p-ACAGUAUUUGGUAUCUGCGC
107mer RNA I	ACAGUAUUUGGUAUCUGCGCUCUGCUGAAGCCAGUUACCUU CGGAAAAAGAGUUGGUAGCUCUUGAUCCGGCAAACAAACCA CCGCUGGUAGCGGUGGUUUUUUUG

Table S2. Oligonucleotide primers and DNA templates used in this study. DNA templates contain a T7 promoter sequence.

Primer	Sequence (5′–3′)
20mer (fwd)	TAATACGACTCACTATTACGGCTCCAGCTACGTAGCG
20mer (rev)	CGCTACGTAGCTGGAGCCGTAATAGTGAGTCGTATTA
25mer (fwd)	TAATACGACTCACTATTACAGCTCAGCCTACGAGCCTGAGCC
25mer (rev)	GGCTCAGGCTCGTAGGCTGAGCTGTAATAGTGAGTCGTATTA
RNA I 107mer (fwd)	TAATACGACTCACTATTACAGTATTTGGTATCTGC
RNA I 107mer (rev)	TCAGCAGAGCGCAGATACCAAATACTGTAATAGTGAGTCGTAT TA
20mer template	TAATACGACTCACTATTACGGCTCCAGCTACGTAGCG
25mer template	TAATACGACTCACTATTACAGCTCAGCCTACGAGCCTGAGCC
RNA I 107mer template	TAATACGACTCACTATTACAGTATTTGGTATCTGCGCTCTGCTG AAGCCAGTTACCTTCGGAAAAAGAGTTGGTAGCTCTTGATCCG GCAAACAAACCACCGCTGGTAGCGGTGGTTTTTTTG

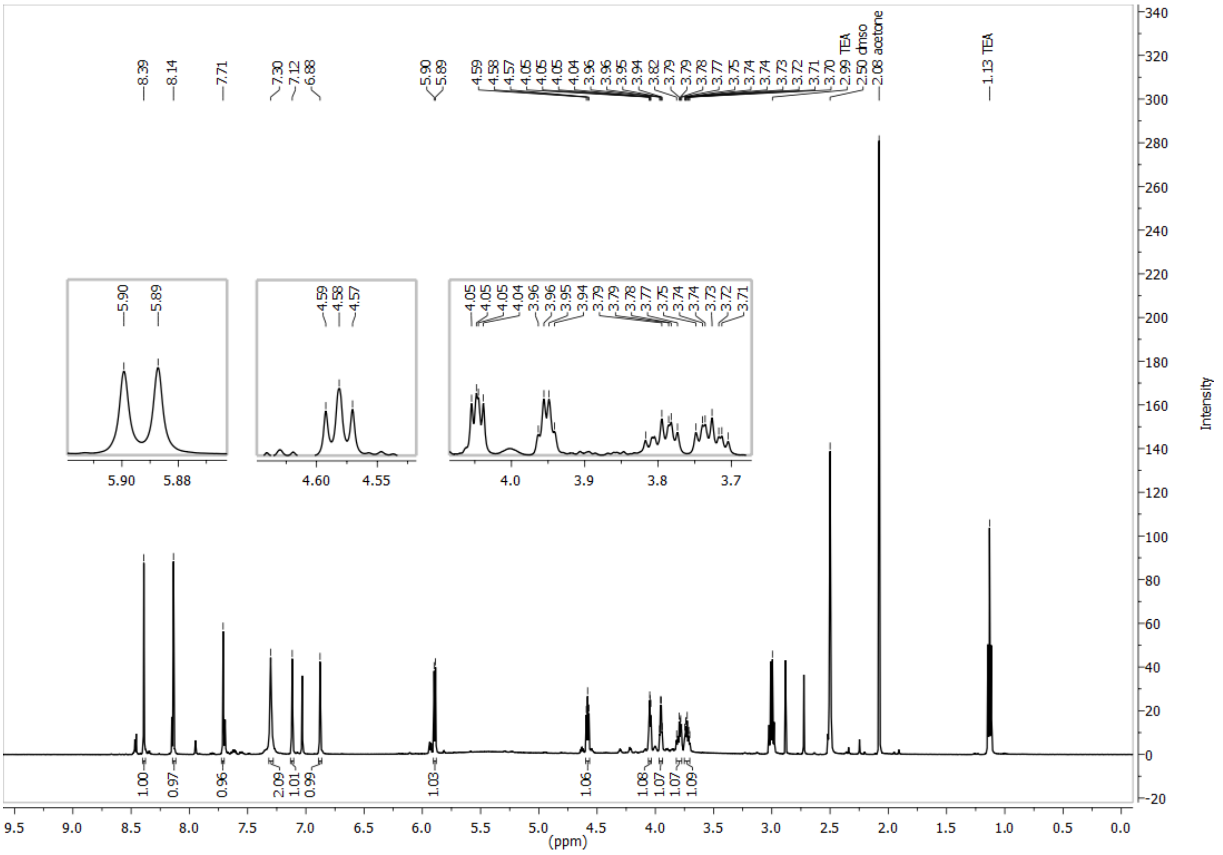

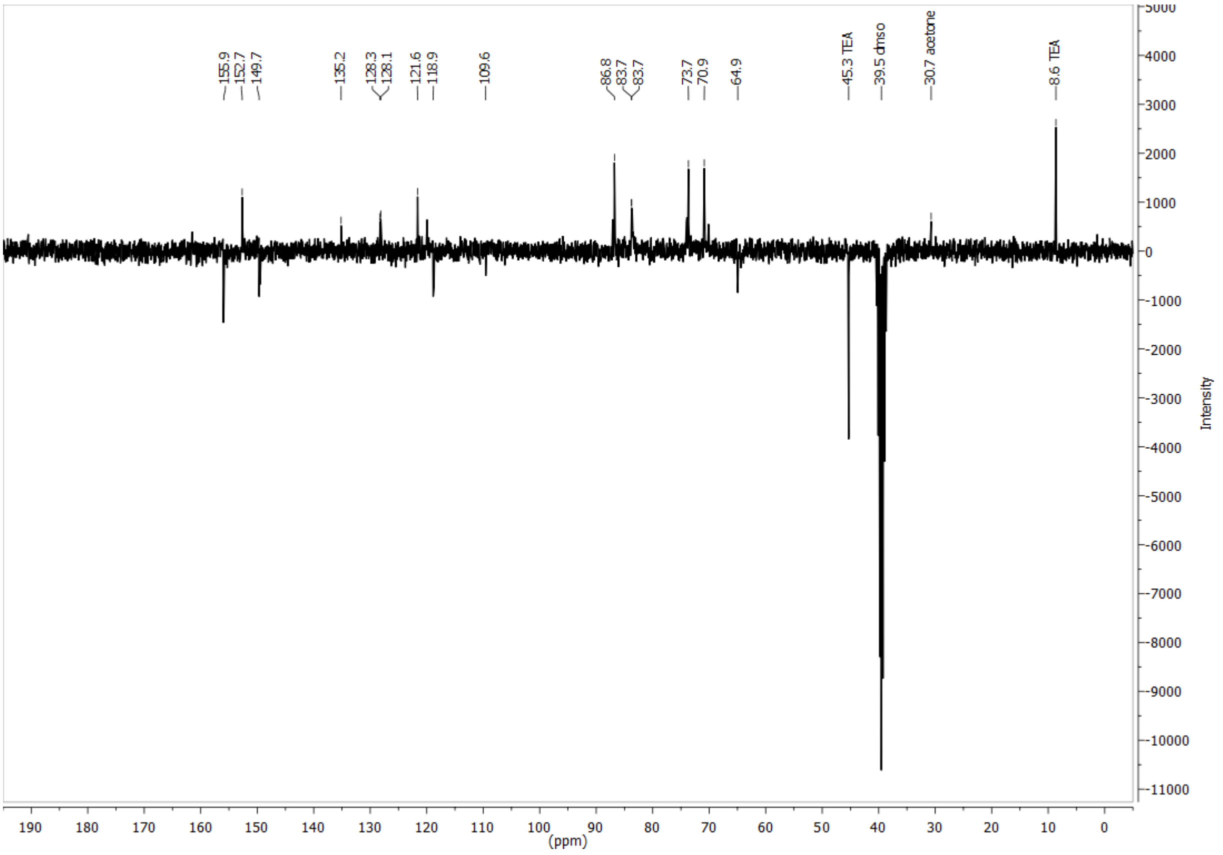

Figure S12. ^{1}H-NMR (top) and ^{13}C-APT-NMR spectrum (bottom) of ImpA.

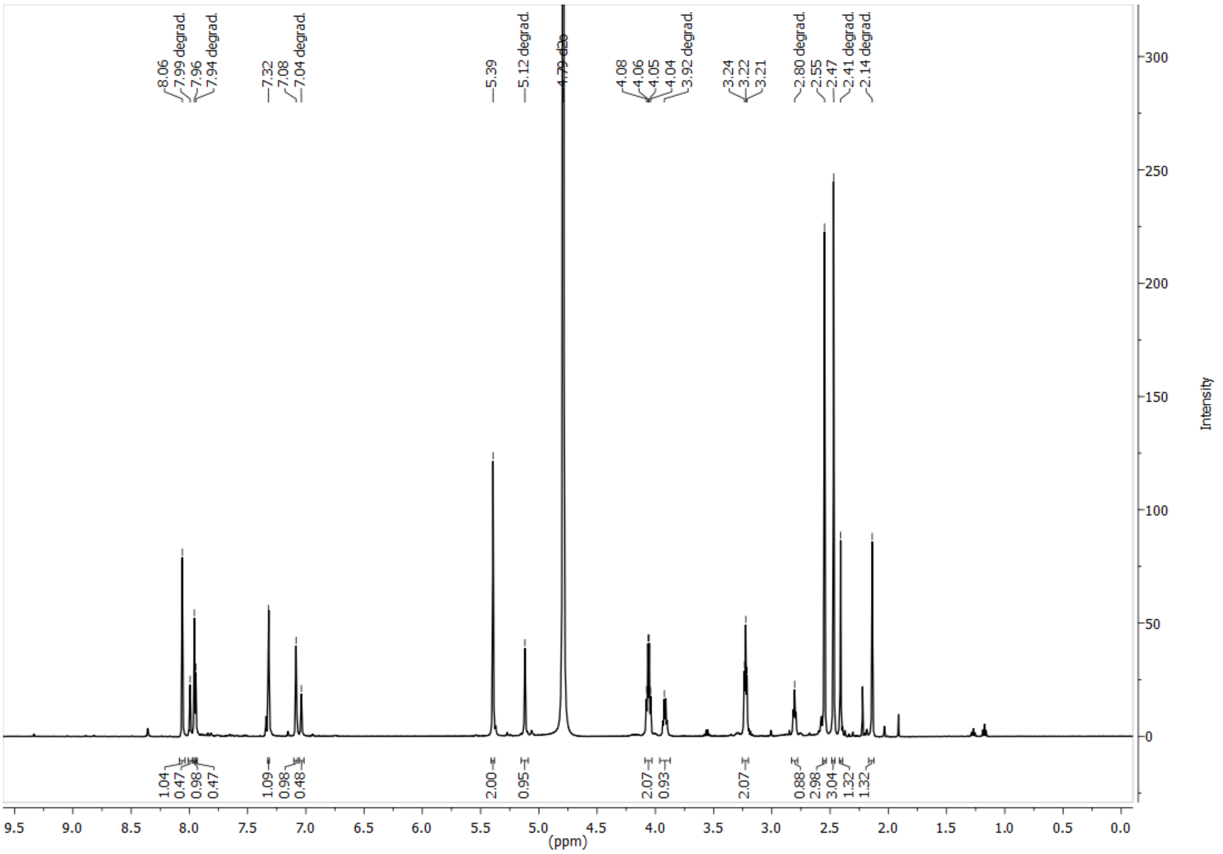

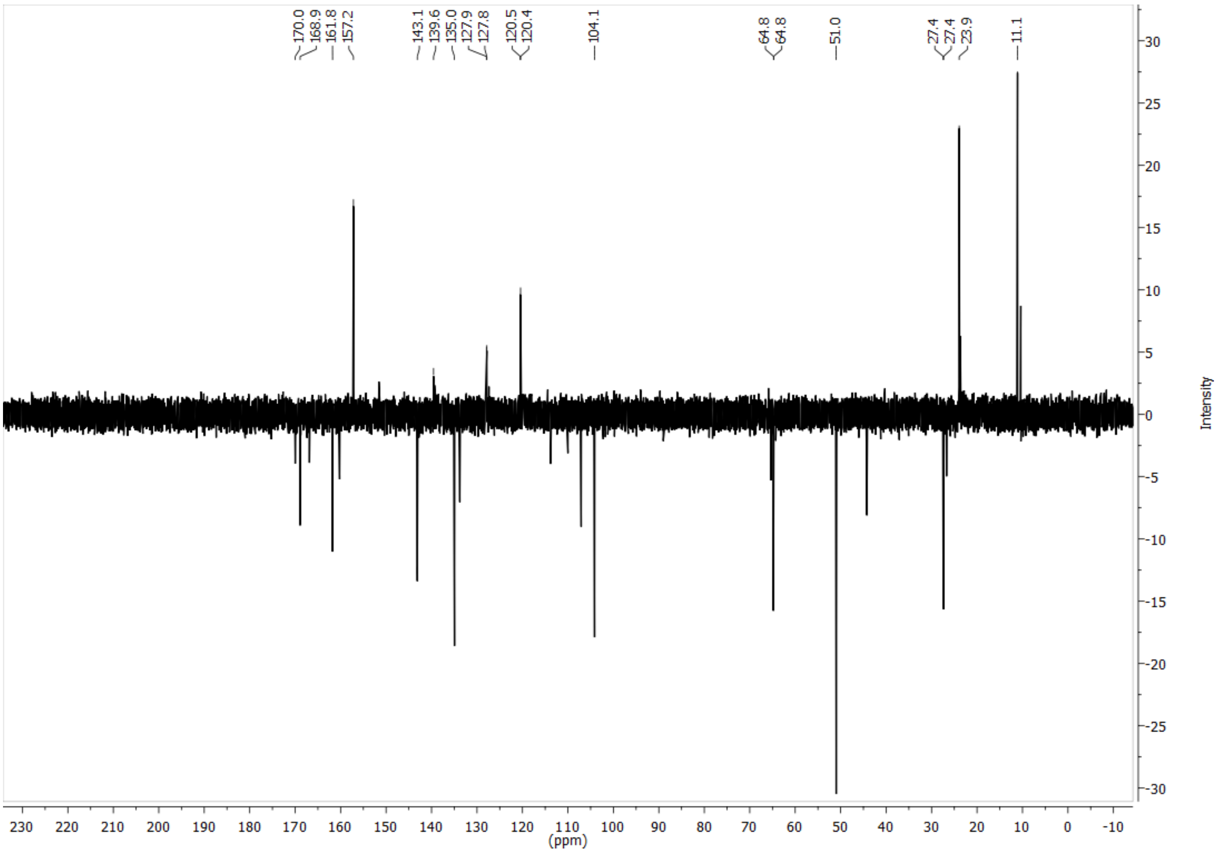

Figure S13. ^{1}H-NMR (top) and ^{13}C-APT-NMR spectrum (bottom) of ImppTh.

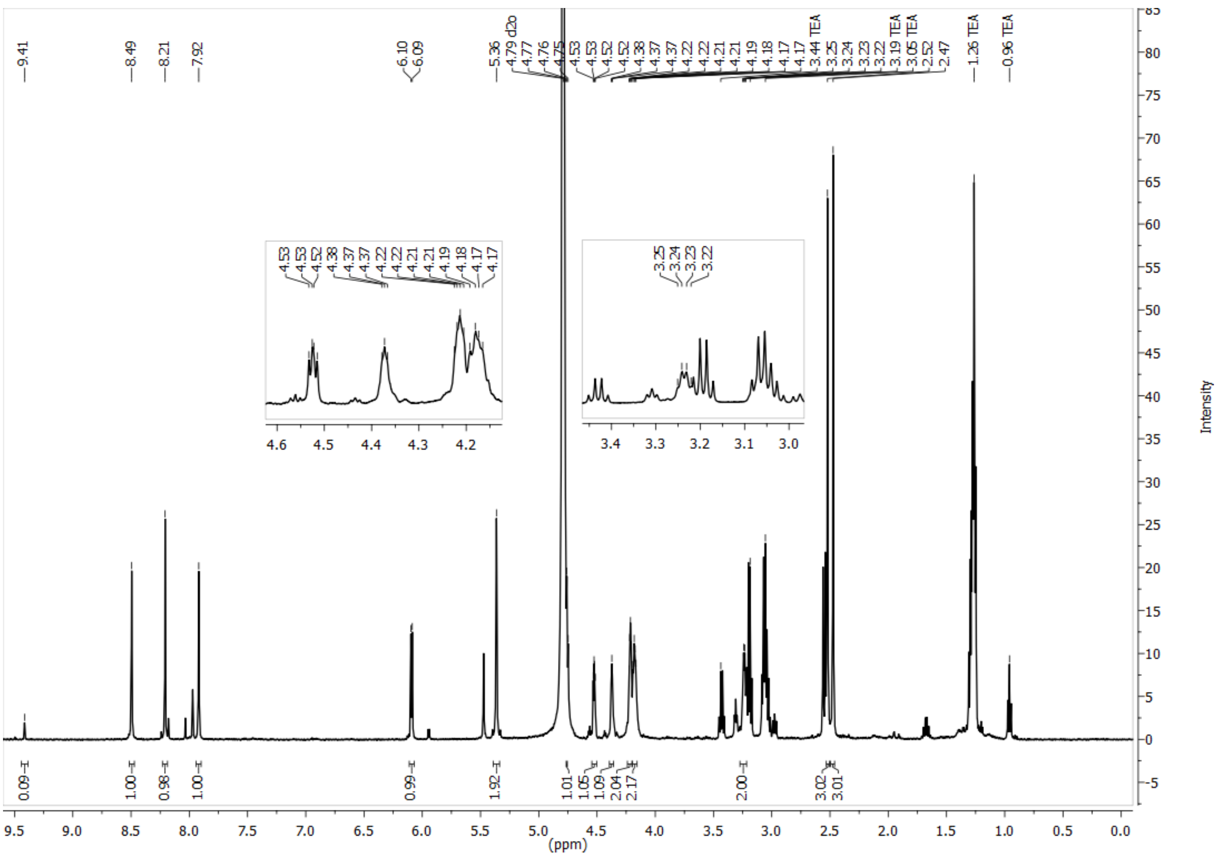

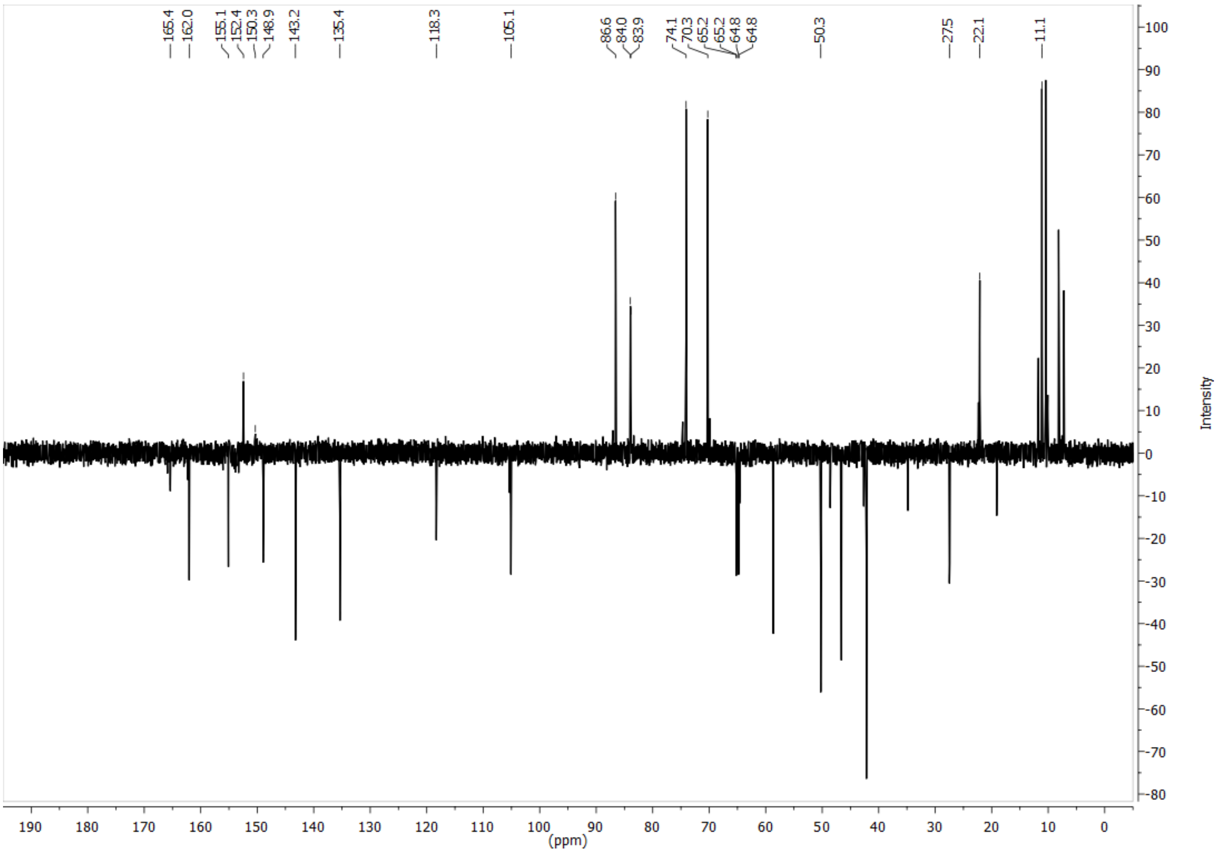

Figure S14. ^{1}H-NMR (top) and ^{13}C-APT-NMR spectrum (bottom) of ThATP.

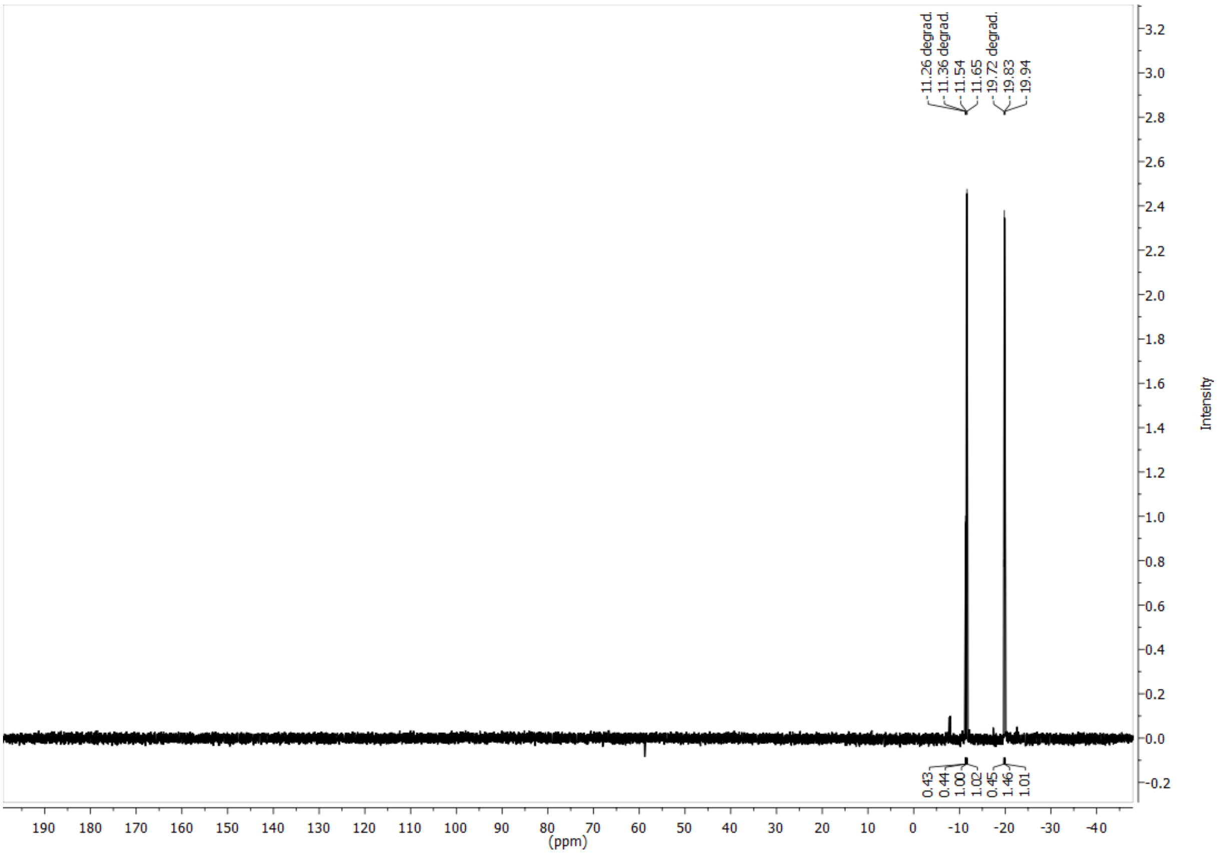

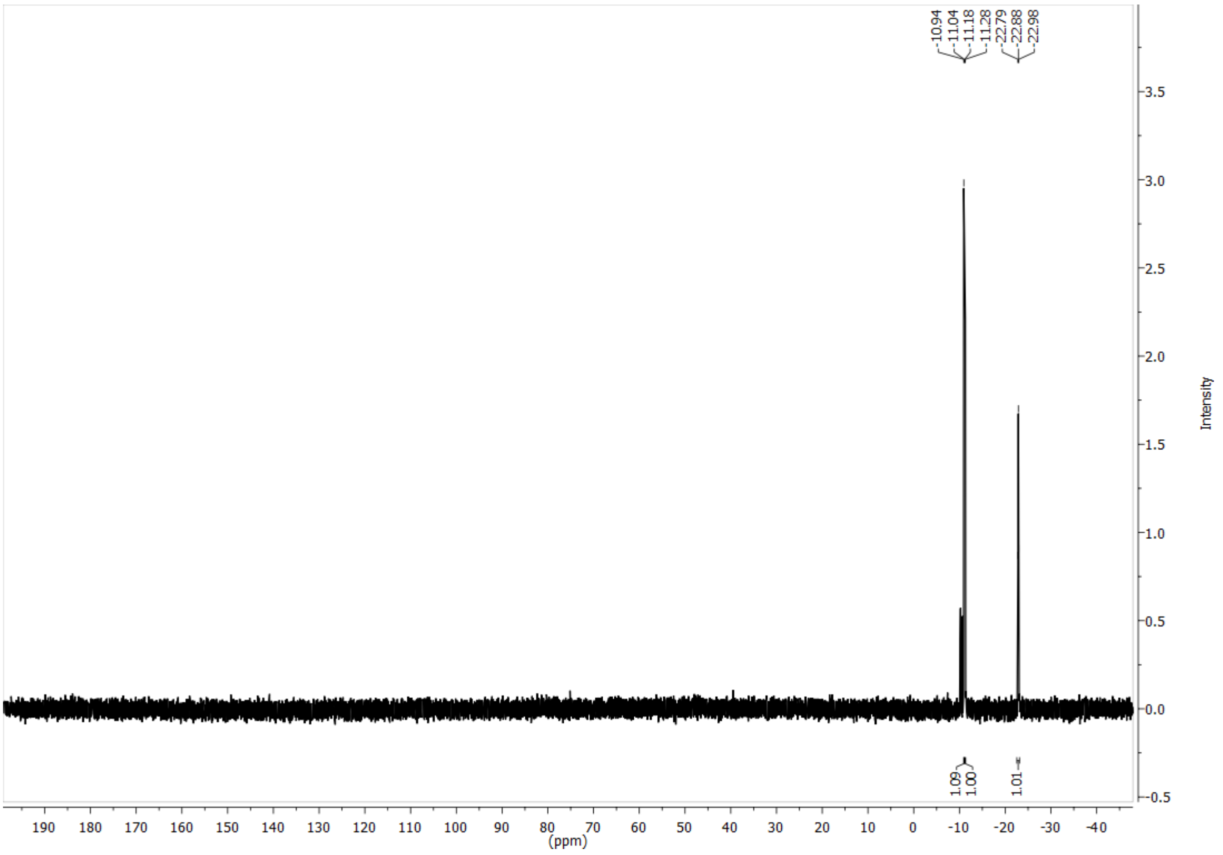

Figure S15. ^{31}P-NMR spectra of ImppTh (top) and ThATP (bottom).

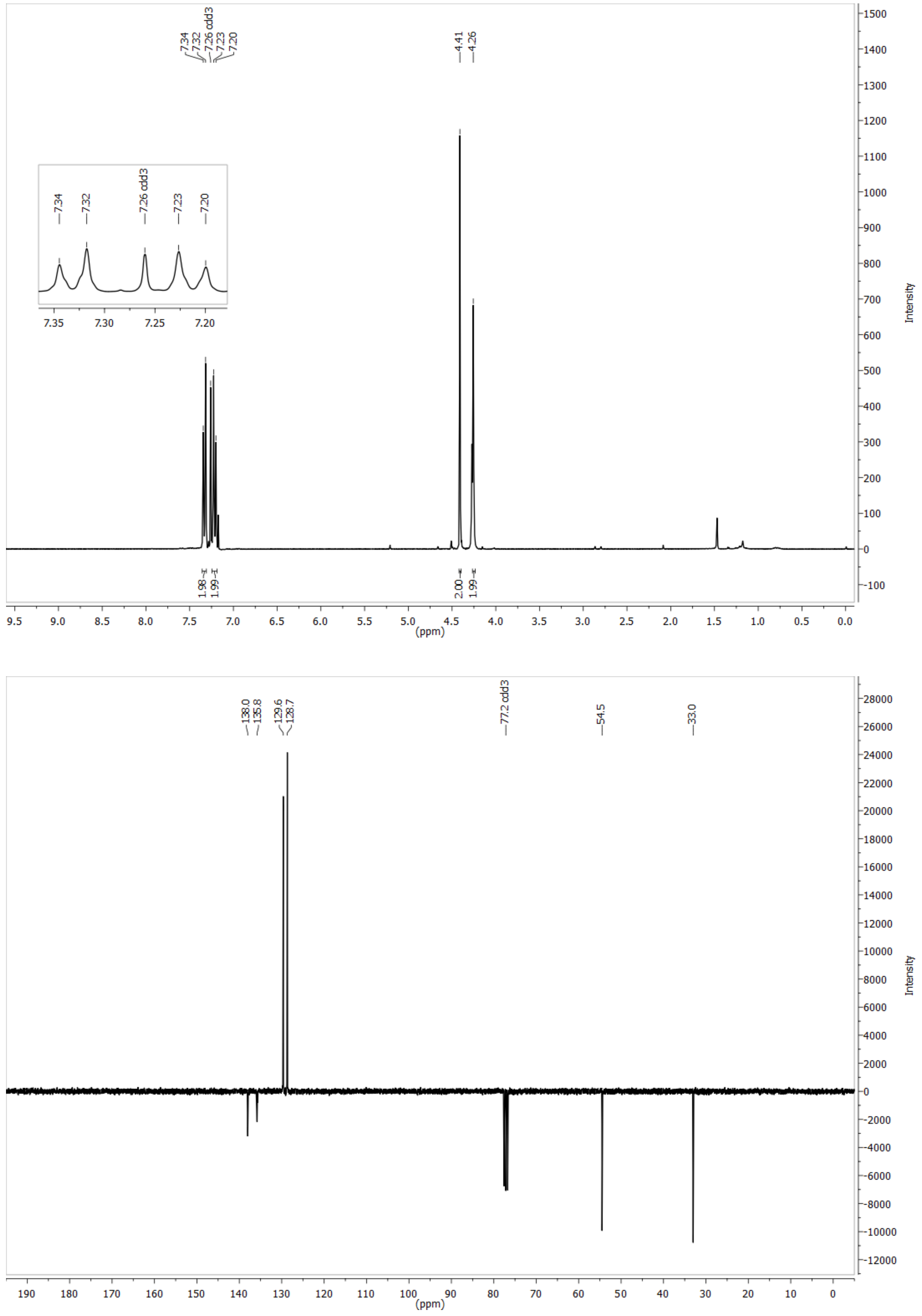

Figure S16. ^{1}H-NMR (top) and ^{13}C-APT-NMR spectrum (bottom) of L01.
